# Understanding repertoire sequencing data through a multiscale computational model of the germinal center

**DOI:** 10.1038/s41540-023-00271-y

**Published:** 2023-03-16

**Authors:** Rodrigo García-Valiente, Elena Merino Tejero, Maria Stratigopoulou, Daria Balashova, Aldo Jongejan, Danial Lashgari, Aurélien Pélissier, Tom G. Caniels, Mathieu A. F. Claireaux, Anne Musters, Marit J. van Gils, María Rodríguez Martínez, Niek de Vries, Michael Meyer-Hermann, Jeroen E. J. Guikema, Huub Hoefsloot, Antoine H. C. van Kampen

**Affiliations:** 1 Amsterdam UMC location University of Amsterdam, Epidemiology and Data Science, Meibergdreef 9, Amsterdam, The Netherlands; 2 Amsterdam Public Health, Methodology, Amsterdam, The Netherlands; 3 Amsterdam Infection and Immunity, Inflammatory Diseases, Amsterdam, The Netherlands; 4 Cancer Center Amsterdam, Amsterdam, The Netherlands; 5 Amsterdam UMC location University of Amsterdam, Medical Microbiology and Infection Prevention, Meibergdreef 9, Amsterdam, The Netherlands; 6 IBM Research Zurich, 8803 Rüschlikon, Switzerland; 7 Department of Biosystems Science and Engineering, ETH Zurich, 4058 Basel, Switzerland; 8 Amsterdam Infection and Immunity, Infectious Diseases, Amsterdam, The Netherlands; 9 Amsterdam UMC location University of Amsterdam, Experimental Immunology, Meibergdreef 9, Amsterdam, The Netherlands; 10 Amsterdam Rheumatology & Immunology Center, Amsterdam, The Netherlands; 11 Department for Systems Immunology and Braunschweig Integrated Centre of Systems Biology, Helmholtz Centre for Infection Research, Braunschweig, Germany; 12 Institute for Biochemistry, Biotechnology and Bioinformatics, Technische Universität Braunschweig, Braunschweig, Germany; 13 Amsterdam UMC location University of Amsterdam, Pathology, Lymphoma and Myeloma Center Amsterdam, Meibergdreef 9, Amsterdam, The Netherlands; 14 Biosystems Data Analysis, Swammerdam Institute for Life Sciences, University of Amsterdam, Amsterdam, The Netherlands

**Keywords:** Immunology, Computer modelling

## Abstract

Sequencing of B-cell and T-cell immune receptor repertoires helps us to understand the adaptive immune response, although it only provides information about the clonotypes (lineages) and their frequencies and not about, for example, their affinity or antigen (Ag) specificity. To further characterize the identified clones, usually with special attention to the particularly abundant ones (dominant), additional time-consuming or expensive experiments are generally required. Here, we present an extension of a multiscale model of the germinal center (GC) that we previously developed to gain more insight in B-cell repertoires. We compare the extent that these simulated repertoires deviate from experimental repertoires established from single GCs, blood, or tissue. Our simulations show that there is a limited correlation between clonal abundance and affinity and that there is large affinity variability among same-ancestor (same-clone) subclones. Our simulations suggest that low-abundance clones and subclones, might also be of interest since they may have high affinity for the Ag. We show that the fraction of plasma cells (PCs) with high B-cell receptor (BcR) mRNA content in the GC does not significantly affect the number of dominant clones derived from single GCs by sequencing BcR mRNAs. Results from these simulations guide data interpretation and the design of follow-up experiments.

## Introduction

The germinal center (GC) plays a crucial role in the adaptive immune response^1–3^. GCs are microanatomical structures found in secondary lymphoid organs and are formed when an adaptive response is initiated. These structures are responsible for a process called affinity maturation during which the affinity and specificity of the BcR for the Ag is improved over the course of several weeks. The GC reaction begins with the activation of a limited number of antigen (Ag)-specific B cells that start to proliferate (clonal expansion) to form the so-called GC dark zone (DZ), as defined by histology staining. During the proliferation of these B cells, now called centroblasts (CBs), their BcR is changed due to somatic hypermutations (SHMs), which increase or decrease the binding affinity of the BcR for the Ag. The CBs differentiate to centrocytes (CCs) and migrate to the GC light zone (LZ) where they collect Ag presented by follicular dendritic cells (FDCs) and, subsequently, interact with T-follicular helper (Tfh) cells to become positively selected to return to the DZ to undergo further rounds of proliferation and SHM. Memory B cells (MBCs) and PCs are output cells (OCs) from the GC. In general, MBCs are of lower affinity than PCs, and are produced mostly at the initial state of the GC reaction, while higher affinity PCs are produced at later stages^4^ although this might be related to the nature of the Ag^5^.

Mammals have an immense immune repertoire comprising B cells and T cells with unique BcRs and T-cell receptors (TcRs) to combat the large variety of Ags. The B-cell repertoire has been estimated to include about 10^15^ members for the naive repertoire although a much smaller fraction of mature B cells is maintained in our body^6^. The diversity of BcR results from several processes that include their development in the bone marrow through somatic recombination of V(D)J genes that encode the receptor and induce junctional diversity, and pairing of different BcR heavy and light chains^7^. Finally, additional diversity is created by SHMs in the GC. The BcR is a heterotetramer composed of two immunoglobulin heavy chains (IgHs) and two immunoglobulin light chains (IgL). Each chain harbors three complementary determining regions (CDRs 1–3) that encompass the most variable parts of the Ab and are responsible for Ag binding. The four BcR framework regions (FWRs 1–4) mostly provide structural support for the CDRs^8–10^.

Immune receptor repertoires in blood or tissue can be profiled using next-generation sequencing technologies^11–14^. These BcR and TcR repertoire-sequencing experiments have been applied for a broad range of applications, including vaccinology, infection, and (auto)immune disorders^15–21^. Typically, the pre-processing of repertoire-sequencing data results in a set of clones and their abundancies in the measured samples. A clone represents a lineage of B cells stemming from the same unmutated common ancestor, which is a naive B cell that initiated a GC reaction as a founder cell. Each clone comprises one or more subclones that differ due to SHM and, therefore, may differ in binding affinity. Each subclone encompasses cells with identical BcRs.

High-abundance clones are a result of Ag-driven clonal expansion and selection in the GC. The top fraction of these clones are generally referred to as dominant clones and provide good candidates for further characterization in terms of binding specificity and affinity^22^, neutralization capacity, and other functional properties^23^. Identified (dominant) clones may also provide good candidates for novel mAb therapeutics such as TNF inhibitors to treat autoimmune disorders^24^, or to neutralize SARS-CoV-2^25^. In addition, they can be used to monitor immune responses during disease or after vaccination^12^. The selection of such candidate B-cell clones is likely to be the most successful when focusing on functional B-cell populations (dominant clones, tissue-infiltrating B cells, plasmablasts, PCs, and MBCs). It is generally assumed that higher abundant clones (dominant clones) have higher affinities due to their Ag-driven expansion and selection in GCs^1,3,26^. Experiments to further characterize repertoires are not always performed because they can be time-consuming and/or expensive. In addition, sometimes one only performs bioinformatics analyses for the interpretation of the repertoire data^27^.

Measuring binding affinities for all hundreds to thousands of clones resulting from repertoire sequencing is, at the moment, infeasible. Since, in practice, one typically selects a specific sequence from a (dominant) clonal lineage as a starting point to create recombinant Ab and, subsequently, measure affinity, one has little information about the variation of binding affinities (or other properties) within a clone. Another point of consideration is the fact that, in the case of RNA repertoire sequencing, the abundance of the clones might be inflated by high immunoglobulin RNA content in PCs. It has been reported that differentiation of B cells into PCs is accompanied by up to a 100-fold increase in immunoglobulin production rate, facilitating the production of secreted Abs^28–33^, which may lead to incorrect identification of the dominant clones.

In previous work, we developed a model of the GC based on ordinary differential equations (ODEs) that suggested that there is only a limited correlation between clonal abundance and affinity^34^. However, in this ODE model we could not analyze individual clones and, therefore, the correlation was based on subclone abundances. Moreover, ODEs provide a continuous approximation to large cell populations and, therefore, low frequent subclones were not adequately represented in this model. In addition, the rate of differentiation into PCs was only based on the affinity of the mother CC. To investigate the reproducibility of the results obtained with the ODE model we set out to modify and extend a much more sophisticated and comprehensive multiscale model representing a single GC that we recently developed^35^. This extended multiscale (eMS) model integrates an agent-based model (ABM) to describe the cellular dynamics, and a system of ODEs representing a core gene-regulatory network (GRN) involved in PC differentiation. Using the eMS model, we aimed to gain a more general insight in the relation between abundance and affinity, the variation of affinity within a clone, and the effect of PCs on the identification of dominant clones by RNA-Seq given their higher BcR mRNA levels. Since we simulate B-cell repertoires generated from a single GC, we also determined to what extent results from these simulations deviate from experimental repertoires established from blood, tissue, and single-cell/single GCs.

Our simulations show that there is a limited correlation between clonal abundance and affinity and, in addition, there is large affinity variability within a clone. Our simulations also suggest that PCs do not have a large influence on the number of dominant clones inferred from RNA-Seq repertoires from single GCs. Finally, as expected, characteristics (e.g., number of clones, diversity of repertoire) of immune repertoires generated by the eMS model deviate significantly from experimental repertoires obtained from blood and tissue. In contrast, repertoires obtained from single-cell sequencing of GC B cells, and from bulk RNA-Seq repertoires from single GCs are in better agreement. Results from these simulations guide data interpretation and the design of follow-up experiments.

## Results

### The extended multiscale model (eMS model)

We recently developed a multiscale model (MS model) of the GC reaction^35^. The MS model integrates a pre-existing ODE model representing a core GRN that drives PC differentiation^36^, and an pre-existing ABM of the GC representing the cellular mechanisms^37,38^. In short, the GC is represented as a 3D sphere of equidistant grid points that also defines the DZ and LZ. CXCL12 and CXCL13 chemokines gradients, resulting from stromal cells in the DZ and FDCs in the LZ, respectively, are imposed on the grid and allow the CBs, CCs, and Tfh cells to preferentially migrate to their respective zones. Founder B cells enter the GC (Supplementary Fig. 1) as CBs, and proliferate and mutate in the DZ, some of them differentiate to CC and migrate to the LZ. There, the CCs compete to interact with the FDCs to capture Ags and present the Ags to the Tfh cells to receive survival signals. If the signals are strong enough, the CC becomes positively selected and comes back to the DZ as a CB. During CBs proliferation, part of the cells differentiate to PCs or MBCs that will leave the GC and have no further function during the simulation. Lack of sufficient survival signals causes the CC to go into apoptosis. The duration of a single-GC simulation is of 504 h (21 days) at a time resolution of 0.002 h.

**Fig. 1:**
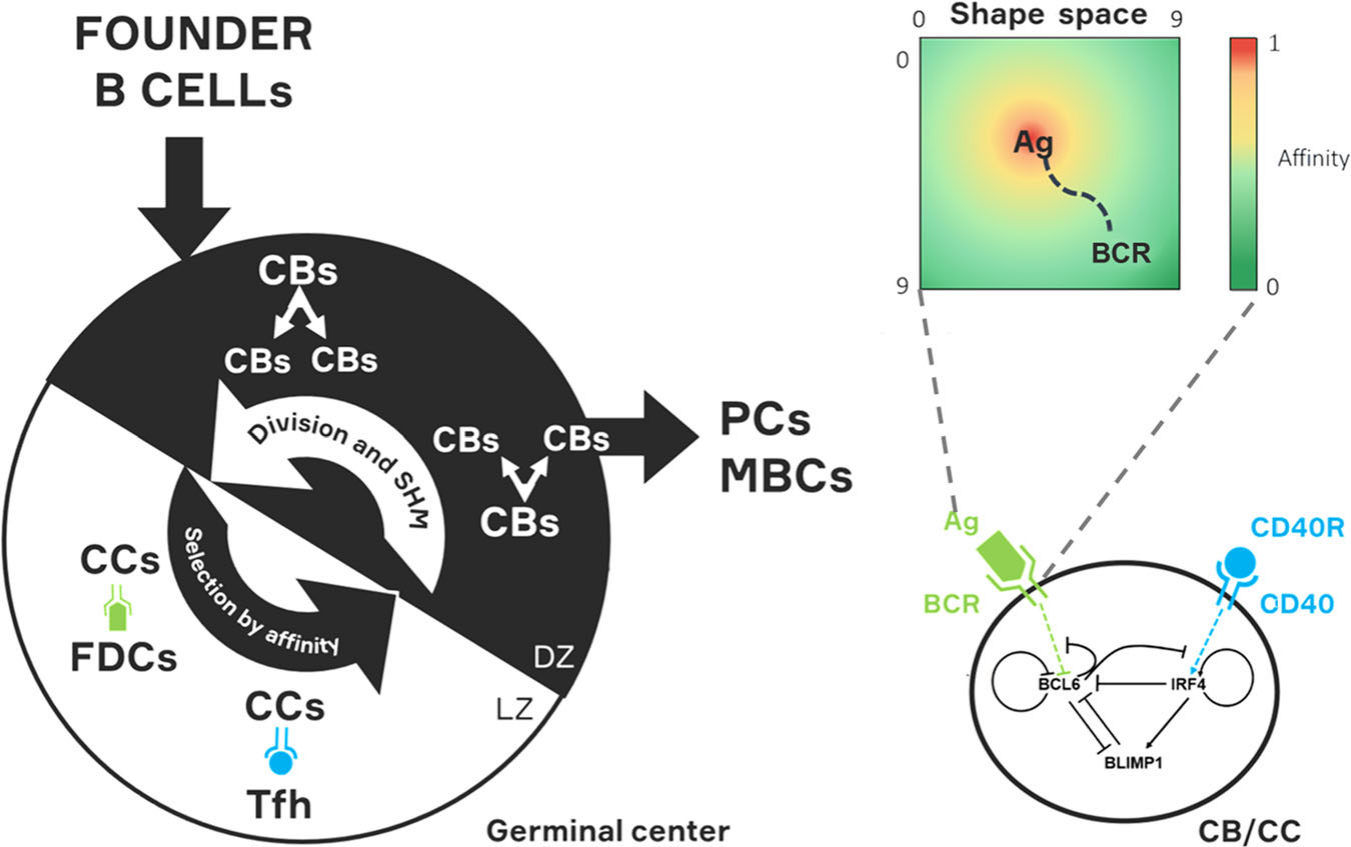
General scheme of our single-GC eMS model. Founder B cells enter the GC and go through a process of division and SHM in the DZ and selection in the LZ, based on the affinity of their BcRs. Each B cell (CB/CC) represented by the ABM embeds a GRN that is affected by BcR and CD40 signaling to drive PC differentiation. The affinity of BcRs can assume values between 0 and 1 and is based on an abstract shape space.

**Fig. 2:**
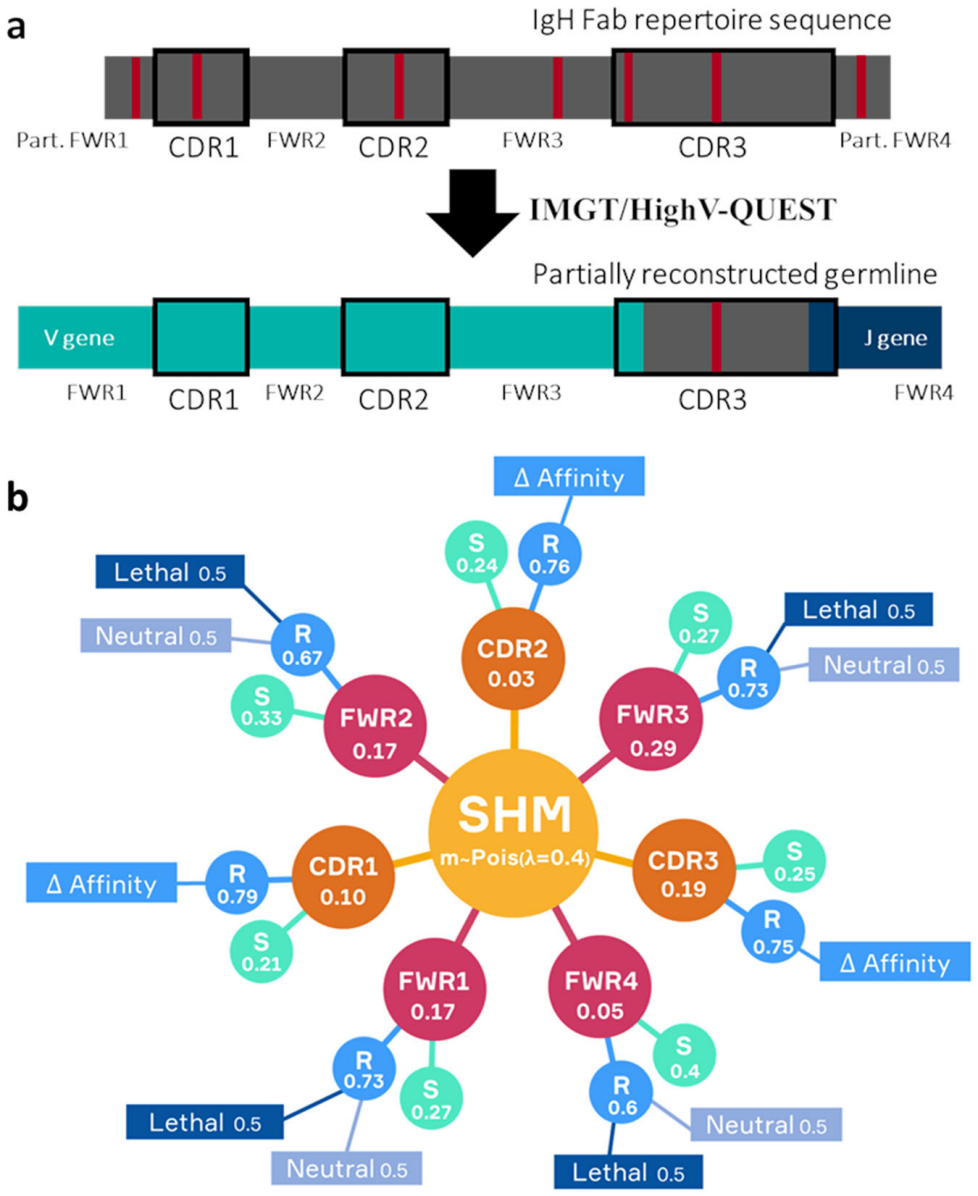
BcR representation and SHM fate tree. **a** BcR sequence reconstruction and representation. From a IgH Fab repertoire sequence from dataset viii, whose FWR1 and FWR4 are partially missing, we infer its V and J genes and the corresponding partially reconstructed germline using IMGT High-VQuest. Red vertical bars represent mutations. The colors on each sequence represent the unknown (gray) and known (turquoise and dark blue) IgH genes. The CDRs appear are marked with black borders. Due to the highly variable nature of the D gene and the junctional diversity, only part of the CDR3 can be assigned to its corresponding germline sequence (i.e., small part corresponding to the V and J germline sequence). The remaining part is taken as is from the repertoire sequence. **b** SHM fate tree. After each cell division, m mutations occur in a daughter cell. This tree shows the probabilities of affecting the different FWR and CDR regions, and the probabilities for making a specific type of mutation (replacement or silent) and its effect (changing affinity, lethal, neutral).

The eMS model (Fig. 1) that we use in this paper is an extension of the MS model. To facilitate the interpretation of BcR repertoires, we implemented a BcR sequence representation for each B cell and OC (Fig. 2a) and now use a SHM fate tree (Fig. 2b and Supplementary Fig. 2) to determine the region and type of mutation. The BcR representation includes the immunoglobulin heavy chain (IgH) and not the light chain (IgL) to be in agreement with experimental repertoire data, which are generally based on IgH. The affinity of the BcRs in the model is based on the distance between the position of the BcR sequence and the position of the optimal BcR in a continuous shape space^37–39^. The shape space does not simulate repertoires specific for an Ag and, therefore, interpretation of the simulations are generalizations^37,38,40,41^. A complete list of model parameters and values is given in Supplementary Text 1.

**Fig. 3:**
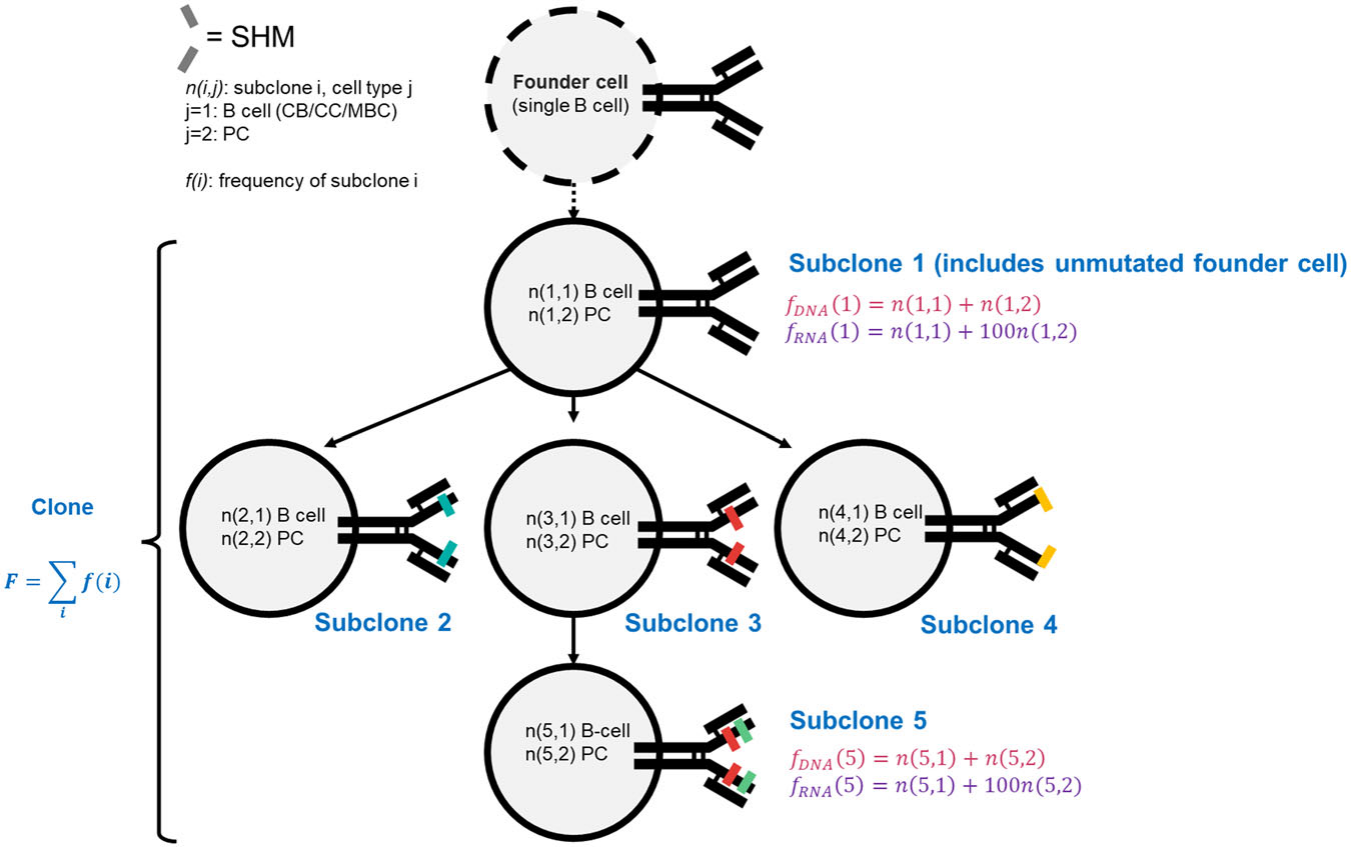
Definition of (sub)clones and their frequencies in the context of a B-cell lineage tree. The founder cell is shown for completeness and is represented by a single cell that enters the GC. Each circle represents a subclone is a group B cells that have the same (mutated) BcR sequence. Different mutations in different positions of the BcR are represented with colored slashes. The five subclones have a common unmutated ancestor and together define a clone. Each subclone is a mixture of CBs, CCs, MBCs, and PCs although a subclone may also consist of a single cell type. The frequency *f*_*DNA*_*(i)* of a subclone is determined by counting the number of cells for each subclone. This frequency can be experimentally obtained with DNA-based repertoire sequencing. Alternatively, frequencies *f*_*RNA*_*(i)* can be determined from RNA-based sequencing but this may artificially increase the subclone frequency if the RNA content of PCs is much higher (a factor of 100 in the figure) compared to CBs/CC/MBCs. The frequency *F* of a clone is the sum of the RNA-based or DNA-based frequencies of the subclones.

The eMS model allows to track the frequencies and affinities of all clones and subclones, (sub)clones, (Fig. 3). The frequency of each subclone equals the number of cells it represents. The clone frequency is the sum of all cells from all subclones. Dominant clones are defined either as the upper 25% of most frequent clones, or as those clones that represent ≥0.5% of the total number of sequences in an experimental repertoire (or cells in a simulation). The latter threshold is based on the analyses of experimental datasets^42^. The D50 index represents the fraction of clones that account for 50% of the BcR sequences.

We performed nine simulations with the eMS model model to generate DNA and RNA-based repertoires. The overall GC dynamics (number of cells, DZ-to-LZ ratio, affinity) for one simulation is shown in Supplementary Fig. 3 and is in agreement with experimental observations^4,43–46^ and with the previous MS model^35^.

#### Comparison to experimental immune repertoires

The number of clones at the end of the nine repeated simulations varies between 4 and 18 at the end (day 21) of the simulation (Supplementary Table 2). The maximum number of clones during the GC reaction is ~200 and equals the number of founder cells (Table 1). The number of clones found in most experimental datasets is orders or magnitude larger compared to the clones produced in our single-GC simulations (Supplementary Fig. 4a). The number of dominant clones from the simulations is more comparable to the experimental data but also is on the lower side compared to it (Supplementary Fig. 4b). Finally, the D50 values resulting from the simulation are larger compared to most of the values obtained from the experimental data (Supplementary Fig. 4c) indicating that in the experiments a smaller proportion of clones account for 50% of the sequences.

**Table 1:** Comparison of different timepoints of our simulations with single-GC data at an unknown timepoint (datasets (viii) and (ix)).

Dataset	Timepoint (hours)	Clones	Dominant clones	Dominant clones (%)	D50 index	Berger–Parker index	Pielou’s evenness index
**Dataset viii**	Unknown, GCs from the same human lymph node	144 (77–591)	30 (20–41)	21 (4.9–28.7)	0.04 (0.01–0.07)	0.17 (0.09–0.33)	0.63 (0.56–0.71)
***Dataset ix***	Unknown, gut-associated GCs from several mice	28 (12–101)	7 (3–12)	28.9 (5.9–53.8)	0.18 (0.05–0.39)	0.24 (0.1–0.45)	0.87 (0.68–0.96)
Simulations	0	0 (0–0)	0 (0–0)	–	–	–	–
Simulations	50	96 (86–114)	54 (45–62)	56 (49–63)	0.20 (0.18–0.22)	0.03 (0.02–0.04)	0.9 (0.88–0.90)
Simulations	100	**180** **(150–198)**	80 (73–89)	44 (41–53)	0.22 (0.19–0.23)	0.03 (0.02–0.05)	***0.93*** ***(0.91–0.94)***
Simulations	150	**128** **(100–153)**	**33** **(23–42)**	**26** **(19–30)**	**0.05** **(0.02–0.07)**	**0.19** **(0.09–0.27)**	***0.74*** ***(0.62–0.79)***
Simulations	200	***59*** ***(43–64)***	18 (12–26)	***35*** ***(27–41)***	**0.04** **(0.02–0.08)**	**0.33** **(0.15–0.62)**	**0.59** **(0.43–0.71)**
Simulations	250	***32*** ***(25–41)***	15 (11–24)	51 (34–70)	0.06 (0.02–0.12)	0.39 (0.20–0.82)	0.58 (0.27–0.77)
Simulations	300	***23*** ***(18–31)***	12 (8–19)	56 (34–73)	0.07 (0.04–0.15)	0.39 (0.19–0.88)	0.58 (0.20–0.80)
Simulations	350	17 (15–25)	11 (6–17)	67 (35–82)	0.09 (0.06–0.17)	0.38 (0.19–0.92)	0.57 (0.16–0.80)
Simulations	400	15 (13–21)	11 (4–15)	71 (31–100)	0.11 (0.06–0.2)	0.40 (0.19–0.94)	0.59 (0.13–0.81)
Simulations	450	13 (10–19)	***9*** ***(3–16)***	75 (30–100)	***0.13*** ***(0.07–0.22)***	0.46 (0.17–0.96)	0.59 (0.09–0.85)
Simulations	504	11 (4–18)	***9*** ***(2–15)***	78 (50–93)	***0.13*** ***(0.07–0.25)***	0.47 (0.15–0.98)	0.60 (0.07–0.85)

Dominant clones were defined as those with counts >=0.5% of the total cell counts. Format (bold; italic bold) denotes the best matching timepoints for datasets (viii) and (ix), respectively.

The number of clones identified in experimental repertoires from the peripheral blood, synovial tissue, and synovial fluid samples are similar but deviate largely from the simulations. This was expected since the number of clones is an accumulation of multiple (past) immune responses involving many GCs, and because peripheral blood contains many naive B cells (singletons). The SPF mouse gaGC results are in line with the simulations at day 21, while the number of clones resulting from the chronic sialadenitis human samples (single GCs) is much higher.

**Fig. 4:**
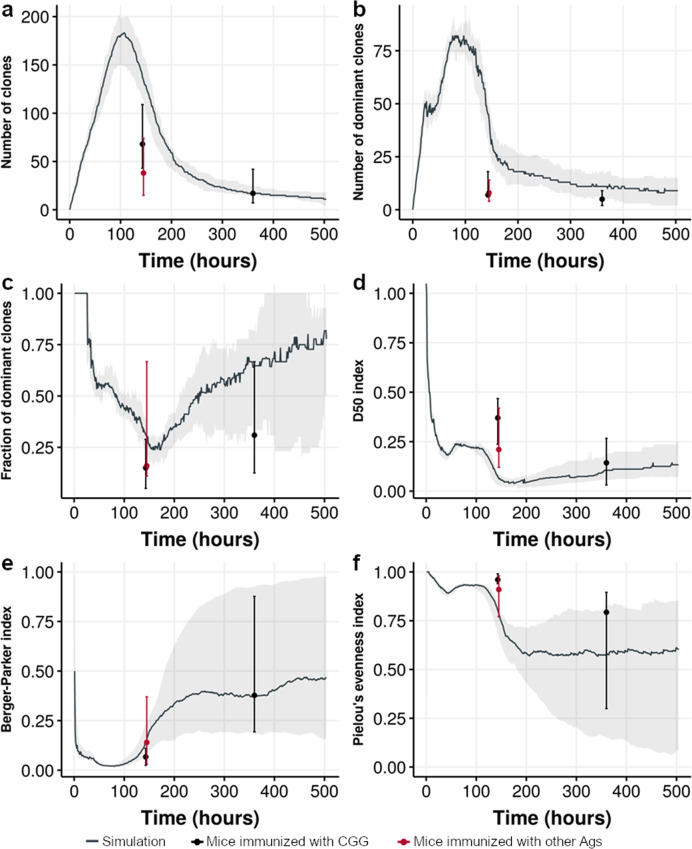
Comparison of nine simulations to dataset x (single GC). Comparison showing the (**a**) number of clones, (**b**) the number of dominant clones, defined as clones accounting for at least 0.5% of the repertoire; **c** fraction of dominant clones; **d** D50 index (the fraction of clones that account for 50% of the BcR sequences); **e** Berger–Parker index (the fraction of cells that belong to the highest abundant clone), and **f** Pielou’s evenness index (measure of the homogeneity of clone sizes ranging from 0, no evenness, to 1, complete evenness). The black curve and gray area represent the median value and observed range from nine simulations. The dot and vertical lines represent the median and observed minimum and maximum range in dataset (x) for the variable at 6 and 15 days after immunization. Vertical black line and dot represent mice immunized with chicken gamma globulin (CGG); vertical red line and dot represent mice immunized with ovalbumin, hemagglutinin or ovalbumin conjugated with 4-hydroxy-3-nitrophenylacetyl hapten).

Since the eMS model represents a single GC, we compared our simulation results at different timepoints with the single-GC datasets (viii to x). The values for dataset (x; single GC from mice) are close to the values in their corresponding timepoint of our simulation results (Fig. 4). Interestingly, there also is some resemblance between the range of minimum and maximum values observed in the experimental and simulated data. Dataset (viii) matches our simulation results at 100–200 h (peak of the GC size^45^ and clonal diversity^47,48^), while dataset (ix) has a larger deviation from our simulations (Table 1). This deviation may be related to the nature of the dataset (steady-state GCs, unknown timepoint, different mice).

#### Progression of clonal size

**Fig. 5:**
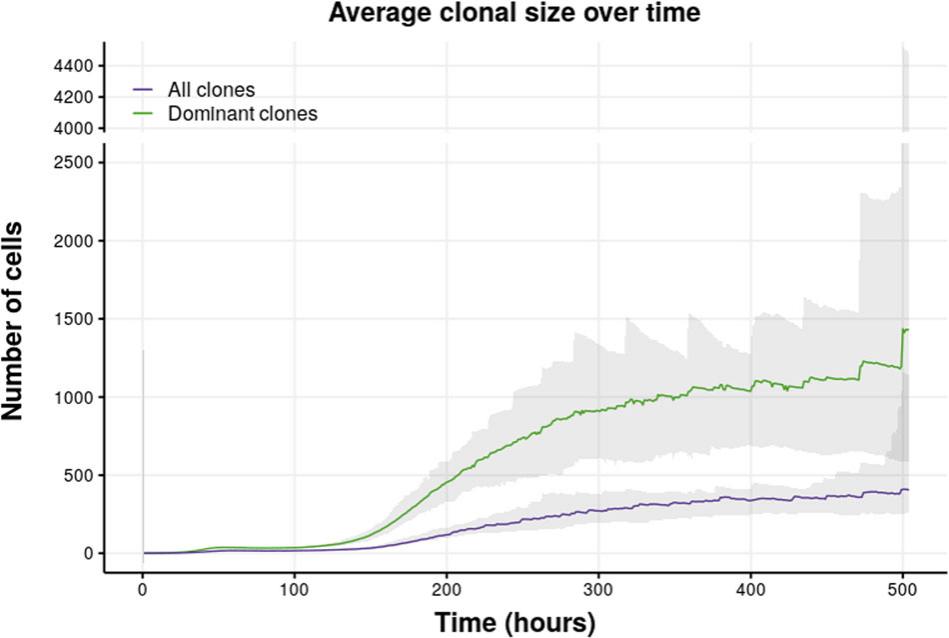
Results from nine simulations showing the evolution of the average clonal sizes of all clones (purple) and all the dominant clones (green) during a 21-day GC reaction. The *y* axis is gapped between 2500 and 4000 cells to facilitate the visualization. Dominant clones were defined as those with abundances higher or equal than the 75th percentile of clonal abundances. The shaded area represents the minimum and maximum of the mean values for each group obtained from the simulations. When the number of clones within a group is low, changes on its clonal composition (a clone may stop belonging to the group because it stops fitting the criteria or because it is outcompeted and removed) lead to big changes in the means.

Figure 5 shows the evolution of clonal size (abundance) during the GC reaction for the dominant and all clones. Dominant clones start outgrowing the average of the population at 120 h of the GC reaction. Supplementary Fig. 5 shows the evolution of the mean clonal sizes of dominant clones and non-dominant clones according to the criteria for dominance. Supplementary Table 2 shows the number of (dominant) clones at the end (day 21) of the GC reaction. Their size increases due to proliferation, while at the same time the number of clones is reduced as a result of their competition (see below).

#### The number of (sub)clones remains at a steady level after the clonal expansion phase but shows a large variability in their affinities

**Fig. 6:**
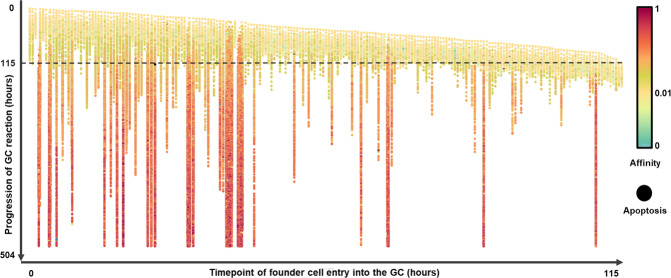
Lineage trees of all founder cells from one representative simulation. Lineage trees of all founder cells from one representative simulation (Simulation 1; Table 1) ordered by the appearance of the founder cell in the GC (*x* axis) and with their daughter cells shown at their time of the appearance in the GC (*y* axis). Approximately 200 lineages each derived from founder cells that enter between *t* = 0 and *t* = 115 h evolve through the GC reaction. See Supplementary Fig. 8 for an individual B-cell lineage. The *x* axis shows the timepoints at which the founder cells enter the GC. The *y* axis shows the evolution of the clones during 21 days (504 h). Colors denote affinity of each individual cell. Apoptotic B cells due to lethal mutations in a FWR (non-functional BcR) appear in black. The initial affinity of the founder cells is 0.01 (yellow). Each lineage comprises a mixture of GC B cells, MBCs, and PCs. Eighteen clones survived in the GC at day 21, while the other clones have been outcompeted and disappeared from the GC reaction.

**Fig. 7:**
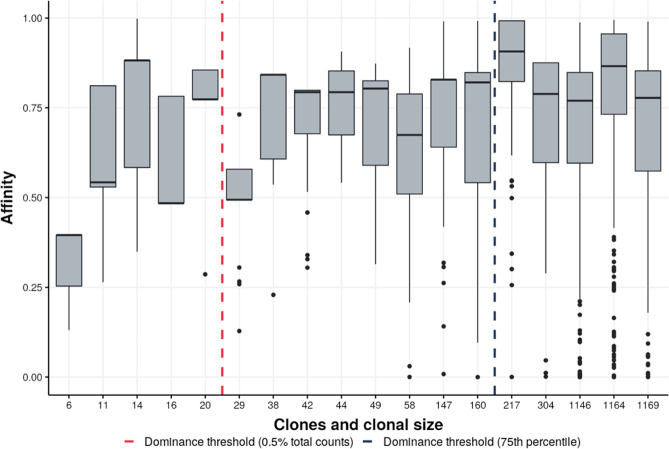
Clonal affinity at day 21. Boxplots representing clonal affinity, showing the large variation in subclonal affinity for 18 surviving clones in the GC at day 21 of a representative simulation (Simulation 1; Supplementary Table 2). The boxplots are sorted and labeled according to the cell count of their corresponding clone. A strong correlation between clonal abundance and affinity that we expected to result from clonal expansion in combination with affinity maturation, is absent. In addition, we also observe highly abundant but low-affinity clones and vice versa. Horizontal line: median. Boxes: 25th and 75th percentiles. Whiskers: 1.5 times the interquartile range. Dots: outliers. The top 5 clones are dominant according to the threshold of ≥=75th percentile of clonal abundances (blue) while the top 13 clones are dominant according to the threshold of ≥=0.5% of the cell counts (red).

We determined the number of clones and subclones during the GC reaction. The GC was seeded with ~200 founder cells (i.e., 200 clones each represented by a single cell). Not all these clones survived the 21-day GC reaction due to clonal competition (Fig. 6). The subclones that descend from founder cells that enter the GC at an early stage generally have more chance to survive because their lineages had more time to increase their affinity and, consequently, outcompete other clones from low-affinity founder cells that entered at later stages. Nevertheless, few clones from late founder cells were able to survive through the full duration of the GC reaction resulting in an average of 12 clones at the end of the simulations (Supplementary Table 2). The number of clones and subclones increases during the initial clonal expansion phase of the GC reaction, after which they slowly decrease while the ratio of subclones per clone slowly increases (Supplementary Fig. 6). The steady number of subclones is in agreement with our previous but simpler ODE model^34^, and is the result of the balance between B-cell proliferation that produces an additional subclone, and SHM that, by definition, removes a single B cell from a subclone and creates a new subclone (different BcR). Consequently, subclones also stay of relatively low abundance (Supplementary Fig. 7). Inspection of the clones at day 21 shows that these are very heterogeneous with respect to the affinity of their subclones. These “high-affinity” clones even harbor B cells of extremely low affinity (Fig. 7).

#### There is a (weak) trend between (sub)clonal abundance and affinity

**Fig. 8:**
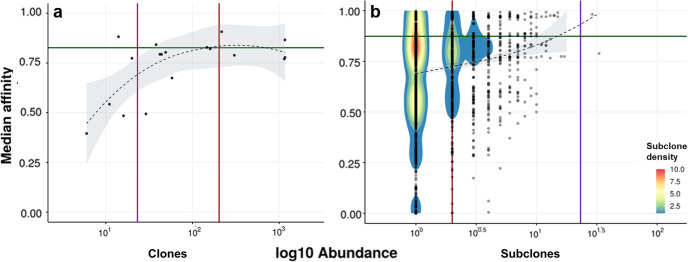
Relationship between abundance and affinity. Relationship between clone (**a**) and subclone (**b**) abundance and median affinity at day 21 of the GC reaction for a representative simulation. Each dot represents a (sub)clone. The horizontal green line denotes the 75th percentile threshold of median affinities. The vertical red line denotes the 75th percentile threshold of clonal abundance. The vertical purple line denotes the 0.5% threshold. The black dotted line denotes a lowess fit. The density map represents the concentration of subclones.

Next, we aimed to determine the relation between (sub)clonal abundance and affinity at day 21 of the GC reaction. The median affinity of each clone was calculated from all its B cells, MBCs, and PCs. The median affinity increases with clonal abundance in the range from one to ~100 but stabilizes for higher abundances. The maximum affinity (1.0) is not reached (Fig. 8a). Using an abundance threshold (>=75th percentile of abundance, or > =0.5% of the total counts) to define dominant clones, and the 75th percentile of the median affinity to indicate high-affinity clones; we observe several low-abundance clones of high median affinity (upper left quadrant Fig. 8a) but also several high-abundance clones of lower median affinity (lower right quadrant). Inspection of the subclonal abundance and median affinity also shows a trend of increasing affinity with abundance but also shows a large variability of affinity for subclones with similar abundance. For example, subclones of low abundance may cover the whole affinity range (Fig. 8b). This variability decreases for the higher-abundance subclones, which are generally of higher affinities.

#### The fraction of PCs does not affect the number of dominant clones

**Fig. 9:**
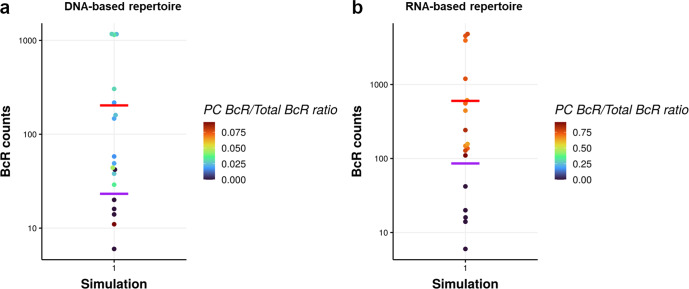
Beeswarm plots in log10 scale of DNA-seq and RNA repertoires. Beeswarm plots in log10 scale of (**a**) DNA-seq and (**b**) RNA repertoires at day 21 of the GC reaction generated by a representative simulation. Each dot represents a clone, some of which are a mixture of B cells, MBCs and/or PCs. In both cases, we find 5 and 13 dominant clones using the 75th percentile or 0.5% threshold, respectively. Dot colors indicate the fraction of PC BcR sequences within each clone, whose range is about a factor of 100 times larger for the RNA-based repertoire. The size of the symbol represents the median affinity of that clone (small symbol: affinity below the 75th percentile). The horizontal lines denote the 75th percentile (red) and 0.5% (purple) abundance thresholds to define dominant clones.

We aimed to investigate if a 100-fold higher immunoglobulin mRNA abundance in PCs leads to erroneous detection of dominant clones in a GC RNA-based repertoire at day 21 of the GC reaction. DNA-seq repertoires are not biased by high BcR mRNA abundance in PCs since for each B cell a single sequence of immunoglobulin gene is generated (Fig. 3). Consequently, in our simulations, we count the number of B cells for each clone to represent the DNA-seq repertoire, while in the RNA-based repertoire each PC is represented by 100 BcR sequences. For the simulated DNA-based repertoires and the simulated RNA-based repertoires this results in a very similar number of dominant clones at day 21 (Fig. 9 and Supplementary Table 2). Most of the clones are a mixture of cell types. Consequently, the RNA-based repertoire increases the frequencies of most clones and, therefore, does not have a large effect on the number of dominant clones (Supplementary Table 2) since it also shifts the threshold accordingly. It does, however, greatly increase the difference in sequence counts between the most expanded clones and the less expanded clones, offering a distorted view. In addition, some minor dominant clones in the DNA-based repertoire may appear as non-dominant in the RNA-based repertoire due to their lack or low abundance of PCs present in the GC at that time point.

The general behavior in the 9 simulations is consistent (Supplementary Fig. 9) and show that not all dominant clones are of high affinity (i.e., median affinity above the 75th percentile). The median affinity of a clone is calculated under the assumption that each cell of the clone contains a single BcR sequence with a specific affinity. Consequently, in a RNA-based repertoire the presence of PCs may skew the median affinity because each PC contributes with 100 sequences to the clone it is part of, compared to a B cell or MBC that only have one single sequence. In some cases, this leads to different high-affinity clones in the RNA-based repertoire.

## Discussion

BcR repertoire sequencing provides information about clones and their frequencies in measured samples. In this work, we used the eMS model to facilitate interpretation of repertoire-sequencing data. In particular, we aimed to study (i) the extent that our simulations represent experimental immune repertoires obtained from blood, tissue, and single GCs, (ii) the relationship between clonal abundance and affinity, (iii) the affinity variability within a clone, and (iv) the extent that the fraction of PCs may affect the identification of dominant clones.

The eMS model is initialized with ~200 clones which number decrease to a much smaller number of clones at the end of the GC reaction. However, the number of subclones remains relatively stable. Most subclones present low cell counts after the clonal expansion phase due to the balance of proliferation and SHM that creates new subclones. The comparison of experimental blood and tissue repertoires to our simulation results at day 21 of the GC reaction (assuming that this is the most representative time point for this) shows in most cases a large difference in the number of clones, the number of dominant clones, and the D50 values. These differences mostly result from the fact that the cellular composition of single-GC repertoires is not the same as the composition of repertoires obtained from blood or tissue, which are composed of different types of B cells, PCs, and MBCs from past immune responses involving many GCs that may be affected by antibody feedback^49,50^. Moreover, not all subclones produced during the GC reaction may travel to blood.

The comparison of our simulation results with single-GC repertoires shows that the simulation is in agreement with dataset (x) in which mice were measured at two timepoints during the GC reaction^48^. The comparison with our simulations indicates that the single-GC repertoire samples obtained from a chronic sialadenitis patient^51^ may be at its peak response (maximum GC size in terms of cells). The steady-state gut-associated mice GC repertoire dataset samples^52^ match different results at different timepoints. There seems to be large variability in the number and size of clones and in the diversity indexes between single GCs, that can be related to the nature of the antigen(s), the lymph node, the species, the duration of the GC response or other biological and experimental factors. Large variability between GCs has also been previously reported^46,53^.

The median affinity of the clones in our simulations shows a weak relationship with clonal abundance, and several high-affinity clones comprise subclones that span a wide range of affinities. This is in agreement with our previous computational ODE model^34^. There are few studies that provide information about the relation between clonal abundance and affinity. For example, Tan et al.^54^ investigated the immune response following influenza vaccination. They selected a limited number of plasmablasts to create recombinant Abs and, subsequently, determined their binding affinity and neutralization capacity for the influenza virus. They found that clones with larger abundances have 10 to 1000-fold higher affinities compared to singleton clones and that within the same clonal family there is up to 43-fold differences in affinity. This is in agreement with our findings, that show important differences between dominant clones and low-abundance clones (e.g., when converting the median affinity of clones in Fig. 7 to experimental affinity^38^ there is a difference in affinity of up to 90-fold) and also indicate that there is not a straight-forward relation between abundance and affinity (Figs. 6 and 7). A recent single-cell study from Mathew et al.^5^ identified a dominant clone with low avidity for HA protein of the influenza A virus. In addition, they found a clone in which two subclones showed a difference of almost a million-fold in affinity. Recently, it has been reported in an OVA-immunized mouse model that affinities measured for plasma cells from blood samples do not correlate with clonal abundance on both a clone and subclone level^55^.

Our simulations confirm that the selection of a single specific subclone (from a dominant clone) for further experimental characterization (e.g., affinity measurement, neutralization potency) does not give a representative picture of a clone and lead to incomplete or erroneous conclusions. In addition, our simulations show that affinity can vary more within a clone than between clones. Both of these observations are in agreement with literature as cited above. For example, although in Fig. 6 the median affinity of the top three dominant clones is greater than 0.75, they host subclones that cover most of the affinity range (0–1). Our simulations suggest that low-abundance (sub)clones might also be of interest since they may have high affinity for the Ag. However, in practice it may prove difficult to select a low-abundance but high-affinity (sub)clone without trial and error.

Our simulations show that a 100-fold increase in BcR mRNA content in PCs does not have a large effect on the number or dominant clones nor on the specific clones that become dominant despite the fact that this has been postulated as a word of caution. However, it does increase the variability in abundance between the dominant clones to some extent. The observation that the number of dominant clones does not largely change is unexpected but mainly due to the fact that also the low-abundance clones represent a mixture of GC B cells, PCs (albeit in lower numbers than more abundant clones) and/or MBCs and, therefore, accounting for RNA content does change proportionally the abundancy of most clones and, consequently, the threshold defining the dominant clones, also shifts. However, our model represents a single GC, where the PCs do not accumulate as they migrate away from it, while in peripheral blood or tissue the proportions of MBCs, PCs and B cells are different and, consequently, “dominant” clones identified from such samples may indeed be the result of high-abundance BcR RNA content in PCs. In addition, it is unknown how many PCs are produced by a single GC and, consequently, whether or not the proportions that result from our simulation are correct. Simulating blood or tissue repertoires would require simulating past and present immune responses (including multiple GC reactions over time^49^, possibly different antigens) and knowing the average life span of B cells, MBCs, and PCs. Further experimental work is required to establish the type of samples and frequency at which the presence of PCs might result in false positive dominant clones.

Although BcR and TcR repertoires are typically generated with bulk RNA or DNA sequencing of blood or tissue samples, there is a progression toward the generation of immune repertoires at the single cell or single-GC level such as the aforementioned study of Nowasad and coworkers who studied B-cell repertoires in gut-associated GCs^52^. Others investigated clonality at the single-GC level^48,56^ without, however, measuring full repertoires. Single-cell sequencing strategies have also enabled the combined transcriptome and immune receptor determination of GC B cells, which may help to improve molecular mechanisms relevant for our model^5,57–60^. One advantage of single-cell strategies is that both the heavy and light chain or alpha and beta chain of the BcR and TcR, respectively, can be determined. For example, the FB5P-seq has been developed and used to determine the transcriptome and receptors (including isotype) of MBCs, PCs, plasmablasts (PB), and sorted GC B cells from human tonsils^61^. Such datasets are expected to increasingly appear in the near future and will help to further validate and improve our model.

Several approaches to simulate repertoire data have been developed^62–68^. SHazaM^65^ introduces random mutations in an input sequence or generates a set of simulated sequences based on a lineage tree using an input sequence as the most recent common ancestor. AbSim^66^ simulates time-resolved repertoires via in silico V–D–J recombination and SHM. immuneSIM^67^ generates B-cell repertoires based on V, D, J germline gene sets and usage; occurrence of insertions and deletions; clonal sequence abundance and SHM (based on AbSim) rate. These generated repertoires can be additionally modified by incorporating motifs, codon replacement and/or changing the sequence similarity architecture. A more recent approach, immuneML^68^ can generate repertoires as randomly generated amino acid sequences, where the amino acids are chosen from a uniform distribution, and can introduce antigen or disease-associated signals in experimental or simulated repertoire datasets.

These methods have a data-driven focus on V(D)J recombination and on imposing SHMs or other variations in the BcR sequences to generate clonal lineages. None of these approaches simulate a GC reaction and, more importantly, none of these considers affinity, PCs, and MBCs as we required for our application.

As such, we consider our model as a first step towards a new type of immune repertoire simulation through the simulation of a GC reaction. Insights from these simulations may facilitate the development of strategies to select (sub)clones for further characterization or development to therapeutic antibodies. However, to simulate repertoires that are more representative for experimental immune repertoires, additional steps have to be taken. First, our model does not represent a specific Ag but instead uses the “shape space” concept to facilitate affinity maturation^38,39,69^. Consequently, we cannot simulate repertoires specific for an Ag and, therefore, interpretation of our simulations are generalizations. More realistic and sophisticated representations might overcome this in the future^41,70^, perhaps even considering the effect that mutations outside the antigen-binding site can have over rigidity, entropic penalty and, ultimately, affinity^71,72^. Secondly, the mechanism for PC differentiation remains to be fully elucidated. Consequently, our implementation of PC differentiation using a small GRN might need to be improved, for example, to account for other transcription factors and cytokines^73,74^. In addition, we did not implement an explicit mechanism for MBC differentiation but rather defined these MBCs as OCs not being classified as PCs^35,40^. Obviously, our approach towards the generation of MBCs and PCs directly affects the number of OCs being generated. This, in turn, may affect the extent to which they affect the identification of dominant clones. Unfortunately, to the best of our knowledge, it is unknown how many MBCs and PCs are produced by single GCs during its entire lifetime, making it difficult to validate the number of OCs produced by our model. However, the aforementioned publication from Mathew et al. allows to make a rough estimate of GC B cells, MBCs, and PCs in mediastinal lymph nodes after infection with the influenza A virus^5^. From Fig. 1 and the underlying data table (kindly provided by Dr. Angeletti, University of Gothenburg) in their paper the fraction of MBCs and PCs from all GC B cells and OCs is about 3.24% and 2.43% at day 14 and 1.7% and 0.7% at day 28, respectively, which is comparable to the total number of OCs we observe at day 21 in our simulations (2.1–2.7%). The implementation of alternative scenarios for MBC and PC differentiation may further improve the model^75,76^. Thirdly, the number of GC founder cells has been estimated to range from two to hundreds with highly diverse early GCs^48,77–79^. The number of founder cells (~200) used in our simulations is within this range but on the high end, although some of the single-GC datasets we have analyzed present up to 600 clones. Reducing the number of founder cells will lead to a delayed GC growth and a lesser number of clones present in the GC at day 21. The diversity of the GC at later stages has been estimated in several studies and ranges from 4 to ~120 clones^48,52,77,80^. Tas and coworkers observed GCs that were predominantly monoclonal but that these are relatively rare^48^. The number of clones at the end of our simulations was between 4 and 18, which is at the lower end of the spectrum. The current model provides little control over the selection pressure to significantly change the number of clones at the end of the GC reaction without disturbing the overall GC dynamics. For example, a simulation with 1500 founder cells resulted in no more than 35 clones at 21 days (data not shown). Therefore, it is worthwhile to expand the model with a mechanism that allows controlling the clonality of the GC to generate a larger variety of repertoires.

This eMS model results in more realistic simulations compared to previous versions thanks to the implementation of the BcR sequence representation on every B cell, MBC, and PC associated to a specific affinity value based on their CDRs and FWRs and to the implementation of a continuous range of affinity values. Our model represents an interesting first step into a new type of immune repertoire simulation given that there is a simulation where OCs are produced and where there is competition over time between clones and subclones.

## Methods

### Repertoire-sequencing datasets

We selected three samples from each of seven blood or tissue repertoire-sequencing studies. The datasets comprise (i) a human single-cell RNA-Seq-based repertoire obtained from three human cell subsets: peripheral blood IgG+ B cells, peripheral plasmablasts after tetanus toxoid immunization, and MBCs isolated after influenza vaccination^81^, from which we used two IgG+ samples and one MBC sample, (ii) a human single-cell DNA-based B-cell repertoire dataset produced in-house from healthy blood donors^82^, (iii) a human bulk RNA-Seq repertoire dataset representing HIV infected patients and (iv) HIV-uninfected controls^83^, from which we used three samples of patients with broadly neutralizing Abs and three samples from the uninfected controls, (v) a human bulk RNA-Seq repertoire dataset from rheumatoid arthritis samples from blood and (vi) one sample from synovial tissue and two from synovial fluid^84^, (vii) a human bulk RNA-Seq repertoire dataset comprising healthy controls and different immune-mediated disorders^15^ from which we used three Crohn’s disease peripheral blood mononuclear cell (PBMC) samples whose CD19 + B cells were sorted. In addition, we selected all the samples from single-GC repertoire-sequencing studies: (viii) an human in-house bulk DNA-based repertoire dataset generated from ten single GCs (two replicates each, singleton and non-shared clones are excluded from the analysis) from a cervical lymph node resected out of a 46-year-old woman suffering from chronic sialadenitis^51^, (ix) fifteen single-cell RNA-Seq samples from mice steady-state specific pathogen-free (SPF) gut-associated GCs (gaGCs)^52^ and (x) a single-cell RNA-Seq dataset of single GCs from mice immunized with chicken gamma globulin (CGG) (eight and twelve samples, taken at days 6 and 15 after immunization, respectively) and from mice immunized with ovalbumin (OVA), hemagglutinin (HA) or ovalbumin conjugated with 4-hydroxy-3-nitrophenylacetyl hapten (NP-OVA) (eleven samples, taken at day 6 after immunization)^48^. For these two last datasets we used the V-CDR3-J assignments provided by their authors, while for the remaining datasets, the BcR sequences the V-CDR3-J assignments were done using Change-O^65,85^. The clonal groups were established according to on V-J stratification, identical CDR3 length and a CDR3 nucleotide similarity of 85% or more. In order to account for sequencing errors, for dataset (viii) only shared clones between replicates are taken into account. In order to account for sequencing depth, for datasets (ix) and (x) we calculated the Chao1 estimator as in their original analyses and used it as a reference.

### Definition of output cells

PC differentiation was mechanistically represented by embedding the core GRN in every B cell, MBC, and PC represented by the ABM of the eMS model. This network comprises three differential equations (Eqs. (1)–(3)) with *p*, *b*, and *r* representing BLIMP1, BCL6, and IRF4, respectively. BCR and CD40 (Eqs. (4) and (5)) represent the signaling strength upon interaction of the B cell with the FDC-presented Ag and the Tfh cell, respectively. Affinity assumes a value between zero and one (see below). The values for the parameters (transcription and decay rates, dissociation constants) are given in Supplementary Table 1. CCs that are positively selected by Tfh cells return to the DZ where they further proliferate and, subsequently, differentiate to a PC if the BLIMP1 level is high enough ([BLIMP1] ≥ 8.10^−8^ M). The PCs leave the GC through the DZ.

1 $$\frac{{dp}}{{dt}} = \mu _p + \sigma _p\frac{{k_b^2}}{{k_b^2 + b^2}} + \sigma _p\frac{{r^2}}{{k_r^2 + r^2}} - \lambda _pp$$

2 $$\frac{{db}}{{dt}} = \mu _b + \sigma _b\frac{{k_p^2}}{{k_p^2 + p^2}}\frac{{k_b^2}}{{k_b^2 + b^2}}\frac{{k_r^2}}{{k_r^2 + r^2}} - \left( {\lambda _b + BCR} \right)b$$

3 $$\frac{{dr}}{{dt}} = \mu _r + \sigma _r\frac{{r^2}}{{k_r^2 + r^2}} + CD40 - \lambda _rr$$

4 $$BCR = bcr0\frac{{k_b^2}}{{k_b^2 + b^2}}$$

5 $$CD40 = affinity \cdot cd0\frac{{k_b^2}}{{k_b^2 + b^2}}$$

For the differentiation of MBCs, we followed a different approach based on asymmetric division of Ag, due to a lack of a clear molecular mechanism underlying this cellular event. It has been shown that Ag internalized by B cells is asymmetrically distributed to the daughter cells during B-cell division^86^. However, a possible effect on B-cell fate was not investigated. Consequently, it was hypothesized that asymmetric division might affect B-cell fate^87^, and this hypothesis formed the basis of the ABM that we use. The original model assumes that CCs positively selected by Tfh cells recycle to the DZ for further proliferation and SHM and that during B-cell division the captured Ag is distributed asymmetrically to both daughter B cells. Subsequently, the Ag-retaining CBs differentiate into OCs, which were not further specified as PC or MBCs. Although there was no direct experimental evidence that asymmetric division determines B-cell fate, the implementation of this mechanism made the computational model in better agreement with B-cell migration patterns observed in experiments of photoactivated B cells^43^. In the eMS model we maintained mechanism of asymmetric division for producing OCs but we distinguished between PCs and MBCs. The MBCs are defined as OCs resulting from asymmetric B-cell division but do not have a high BLIMP1 level. This approach towards PC and MBC differentiation ensured agreement with an experimentally observed temporal switch in which lower affinity MBCs are mainly produced at the initial phase of the GC, while higher affinity PCs are produced after the peak response^4^.

### Influx of founder cells

Following experimental observations^48^ we initiated the GC simulations with ~200 founder B cells each that enter during the initial phase of the GC reaction^38^. Accordingly, founder B cells enter the GC with a probability p(influx).

6 $$p\left( {{{{\mathrm{influx}}}}} \right) = \frac{{\mu \cdot {{\Delta }}t}}{{1 + e^{\frac{{t - \alpha }}{\beta }}}}$$

With Δ*t* = 0.002 h corresponding to the time resolution of the ABM, *α* = 96 h representing the time point at which influx stops, *β* = 6 h and represents the rate smoothness, and *μ* = 2 cells/hour represents the inflow rate. Integration of this equation shows that this leads to approximately 200 founder B cells (Supplementary Fig. 1). At time point *t* = 0, this gives a probability of *P* = 0.004. In the first hour, this leads to an influx of ~0.004*(1/Δ*t*) = 2 cells.

### BcR sequence representation

We built a set of partially reconstructed germline heavy-chain V(D)J sequences from the BcR repertoire of two single GCs (dataset viii) using IMGT High-VQuest^88,89^ (Fig. 2a). Due to the high variability of the CDR3 (average length of ~48 nucleotides^90^), it is difficult to correctly identify the short D gene (average length of nearly 24 nucleotides), or to precisely determine the junctional diversity^91^. Consequently, we identified the V and J sequences, which include part of the CDR3 region. The remaining CDR3 part will remain identical to the experimental sequence. Due to the placement of primers in the FWR1 and FWR4, part of these regions is missing. We fully reconstruct these regions in the reconstructed sequence. For the reconstructed sequence, we annotated the four framework regions (FWR) and the three complementary determining regions (CDRs). Each founder B cell is associated with a BcR nucleotide sequence that is randomly selected (without replacement) from this set of unique V-CDR3-J sequences. This BcR sequence includes the immunoglobulin heavy chain (IgH) and not the light chain (IgL) so our results can be easily extrapolated, as most clonal repertoire datasets and analyses only study the IgH. During the GC reaction this sequence is mutated (see below) and inherited by the (founder) B-cell progeny.

### Affinity and shape space

The calculation of the binding affinity between a membrane-bound BcR and a specific Ag requires computer modeling approaches such as molecular dynamics and, therefore, are very compute intensive^92^. This makes these methods impractical for use in computational simulations of the GC because for each (combination of) SHM the binding affinity would have to be recalculated. Therefore, in our computational model, affinities assume values between 0 and 1, which are based on an abstract “shape space” that was initially proposed by Perelson et al.^39^ to represent the complementarity between the BcR and Ag protein shape. In earlier versions of the GC ABM this space was defined as a 4-dimensional discrete grid comprising 10,000 positions in which the Ag assumed a fixed position and the BcR moved through this space as a result of SHM^38,39,69^ (Fig. 1). The distance (L1-norm) in this space between the Ag and BcR represents the number of mutations required to acquire the maximum affinity, and is converted to an affinity value using a Gaussian weight function. Consequently, affinity values in the model cannot be compared to experimental binding affinities and do not represent binding to a specific Ag^37,38,40,41^. The shape space approach to determine affinity values only facilitates the process of affinity maturation. However, the Gaussian weight function is parameterized to be in agreement with experimental data regarding the number of mutations and observed affinity fold changes. In our extended model we changed to a continuous shape space grid because the discrete grid can only provide 25 different affinity values, which is too limiting in our case where we represent the BcR as nucleotide sequences and, consequently, needed to accommodate much more different affinity values as result of SHM. Our continuous shape space has a length of ten (arbitrary units) for each dimensions and allows an infinite number of affinity values between 0 and 1. Upon SHM, the BcR will move in a randomly selected dimension. with a step size *s* sampled from a normal distribution with a mean of one and a standard deviation of 0.1 (*s* ∼ *N*(*μ* = 1, *σ* = 0.1)). Subsequently, the L1-norm (Manhattan distance) between the Ag and BcR is calculated and converted to an affinity value using the Gaussian weight function. Each step in the shape space may increase or decrease the distance from the Ag and, consequently, decrease or increase the affinity by an amount according to the step size.

### Fate of somatic hypermutations

Twenty-four hours after the initiation of the GC reaction, SHM is switched on with a rate of 10^−3^ mutations per base pair per B-cell division^93,94^. Although both the BcR heavy and light chain are important in Ag binding, we simplified our approach by only considering mutations in the heavy chain, in agreement with our BcR sequence representation. Since the average length of a BcR heavy chain is ~400 nucleotides resulting in 0.4 heavy-chain mutations per cell division, we modeled the number of mutation (m) as *m* ∼*Poisson* (*λ* = 0.4). This results in one or more mutations in ~33% of the cell divisions (Supplementary Fig. 2). Each mutation affects a specific FWR or CDR region, which is selected probabilistically using a SHM fate tree (Fig. 2b). This decision tree also determines the type of mutation (replacement or silent), and, according to its type and region, its effect (changes affinity, is lethal or is neutral). Once a region is selected then within this region, we randomly replace a nucleotide and check from the corresponding amino acid sequence if the type of mutation (replacement, lethal, neutral, or silent) agrees with the selected mutation type from the tree. If not, a different nucleotide will be selected until the process is successful or all combinations are exhausted, in which case a new position is randomly selected from the region and the nucleotide replacement restarts. We do not take into account mutation hot/cold spots^95^. Our decision tree is an extension of the fate tree previously constructed from experimental data but which does not distinguish between the individual FRW and CDR regions^62^. We extended this tree to represent all seven FRW/CDR regions. The probabilities in the tree were obtained from sequence data from non-expressed (non-functional) κ light chain transgenic mice immunized with nitrophenyl (NP)^95^ resulting in mutation patterns in the absence of Ag-driven selection pressure. Preferably, these probabilities should be estimated from (human) heavy-chain non-functional sequences but to the best of our knowledge, such data are currently not available. Therefore, we assumed that these probabilities are representative for the human heavy chain. We also assumed that only CDR replacement mutations affect affinity, not considering the effect that mutations outside the antigen-binding site can have over rigidity, entropic penalty and, ultimately, affinity^71,72^. The fate tree does not account for key or blocking mutations^96^. The probabilities for lethal mutations are taken from the original fate tree^62^. A lethal mutation will set the affinity of the CB to zero and will make it go into apoptosis in the DZ^97^. Compared to the original ABM, the use of the fate tree changes the probability of a SHM taking place and the probability of the affinity of a cell changing when a SHM takes place. In the original model, a maximum of one SHM per daughter cell could happen; the SHM probability was set to 0.5 resulting in one mutation, on average, during each B-cell division, and said mutations always leaded to a change in affinity. In contrast, in the eMS model more than one SHM per daughter cell can happen, the probability of at least one SHM happening is of 0.33 and the probability for a mutation to change the affinity is of [(0.1*0.79) + (0.03*0.76) + (0.19*0.75)] ≈0.244 (Fig. 2b). If we take into account the different probabilities for a daughter B-cell of having one, two, or three mutations per division (Supplementary Fig. 2), this leads to an approximate total probability of (1*0.27 + 2*0.05 + 3*0.01)*[(0.1*0.79) + (0.03*0.76)+(0.19*0.75)] ≈0.1 of a mutation changing affinity taking place in a daughter B cell after division.

To ensure the consistency of affinity values across the mutated sequences during the GC reaction, we store each combination of affinity and BcR sequence in a database (Supplementary Text 1).

### Simulations

We performed nine simulations to generate DNA and RNA-based repertoires with our multiscale model, using different random seeds to account for the stochasticity of the ABM. The initial affinities of the founder cells are set to identical but a low value of ~0.01 corresponding to a Manhattan distance of approximately 6 between BcR and Ag in the shape space. From each simulation a DNA-based BcR repertoire was generated at 21 days of a single-GC reaction. In this DNA-based repertoire the number of BcR sequences reflect the relative abundances of the GC B cells, MBCs, and PCs, i.e., each BcR represents a single cell. From the DNA-based repertoire, we generated a RNA-based repertoire by assuming a BcR mRNA abundance in PCs that is 100-fold higher compared to CBs/CCs/MBCs. Although our analysis of IgH expression in single-cell RNA-Seq datasets from human tonsils (Supplementary Fig. 10) points to a scenario where the median value of immunoglobulin production rate is ninefold higher in PCs than in GC B cells and MBCs, our assumption is a more extreme scenario in line with the previously reported increase in some cases of up to 100-fold in the immunoglobulin production rate^33^. Consequently, we multiply the frequency of each PC subclone per 100 (Fig. 3). Effectively, we generate 100 BcR sequences for each PC. Consequently, this may raise the frequency of the PC subclone and its clonal group above the threshold that defines dominant clones, resulting in its false assignment as a dominant clone (Supplementary Fig. 11A). To determine the relation between (sub)clonal abundance and affinity we performed a locally weighted scatterplot smoothing (Lowess)^98^.

### Comparison to BcR repertoires

We compared the number of clones, the number of dominant clones and the D50 index of our results at 21 days with the repertoires from blood, tissue or single GCs measured at unknown timepoint (datasets i to viii). We extended this comparison for the single-GC datasets (datasets viii to x), including the clone dominance using the Berger–Parker^99^ index (the fraction of cells that belong to the highest abundant clone), the homogeneity of clone sizes using the Pielou’s evenness index^100^ (a value between 0, dissimilar, and 1, similar) and the fraction of dominant clones obtained from our simulations at different timepoints.

### Reporting summary

Further information on research design is available in the Nature Research Reporting Summary linked to this article.

## Supplementary information

Supplementary Information

Reporting Summary

## Data Availability

Simulation parameters and setup details are available in Supplementary Table 1 and GitHub [https://github.com/EDS-Bioinformatics-Laboratory/GC_ABM_SHM_network]. Individual simulation results generated and analyzed during the current study are available at 10.5281/zenodo.7642721. Dataset (ii), analyzed during this study is available at NCBI GEO under the accession number GSE196820. Datasets (v) and (vi), analyzed during this study, are available under the accession number PRJNA822925. Dataset (viii), analyzed during the current study, is available from J.G, and its processed data are deposited on the VDJ server under UUID 8899006209436478995-242ac118-0001-012. The remaining datasets are available from their original publications.

## Code availability

Source code and simulation parameters used for this work are available from GitHub [https://github.com/EDS-Bioinformatics-Laboratory/GC_ABM_SHM_network]. Library information can be found in Supplementary Text 2.
