# Supplementary material for: Understanding repertoire sequencing data through a multiscale computational model of the germinal center
Source: NPJ Syst Biol Appl. 2023 Mar 16;9:8. doi: 10.1038/s41540-023-00271-y (PMC10019394; doi:10.1038/s41540-023-00271-y)
Supplement: Supplementary file 1 — Supplementary Information [file 41540_2023_271_MOESM1_ESM.pdf]

Understanding repertoire sequencing data through a multiscale computational model of the germinal center

Rodrigo García-Valiente[#], Elena Merino Tejero[#], Maria Stratigopoulou, Daria Balashova, Aldo Jongejan, Danial Lashgari, Aurélien Pélissier, Tom G. Caniels, Mathieu A. F. Claireaux, Anne Musters, Marit J. van Gils, María Rodríguez Martínez, Niek de Vries, Michael Meyer-Hermann, Jeroen E.J. Guikema, Huub Hoefsloot, Antoine H.C. van Kampen[*]

[*] a.h.vankampen@amsterdamumc.nl

Supplementary Information

Maintaining consistency among mutations

Supplementary Text 1. To ensure consistency of affinity values across the mutated sequences during the GC reaction, we store each combination of affinity and BcR sequence in a database. If a sequence is mutated, we determine its fate and, subsequently, update its affinity according to the following rules:

1. For a silent mutation in a region, we randomly select a nucleotide position, arbitrarily change said nucleotide and check that the corresponding amino acid is not changed. Otherwise, a different nucleotide position is selected.
2. For a lethal mutation in a FWR (replacement) we randomly select a nucleotide position and arbitrarily change the nucleotide at this position in a way that also changes the aminoacidic sequence. Subsequently, we check our database to ensure that this mutation was not already associated with a different type of mutation (neutral) from an earlier time point. If not, then the mutation is accepted we set the affinity of the B cell to zero and we update the database. Otherwise, we select a different nucleotide or we select a different position if none is viable until the process is successful. Finally, the database is updated.
3. For a neutral mutation in a FWR (replacement) a nucleotide position is randomly selected, said nucleotide is changed into a different nucleotide in a way that changes the corresponding aminoacid. The database is checked to confirm that the selected mutation was not associated with a lethal mutation at a previous time point. If not, then the mutation is accepted and stored as neutral, otherwise a new nucleotide is selected until the process is successful. Finally, the database is updated.

4. For an affinity changing mutation in a CDR (replacement) we randomly select a nucleotide position and change it to a different nucleotide such that this gives a change in the aminoacidic sequence. If a combination of identical CDR1, CDR2, and CDR3 amino acid sequences is present in the database then the affinity of the new daughter B cell is set to the affinity assigned to this combination in the database, otherwise a new affinity is determined from the shape space, based on the position of its mother cell or, in the case of back mutations, of its founder cell. Finally, the database is updated. It is important to take into account that subclones with different combinations of CDRs can have descendant subclones that share the same combination of CDRs between them (converge). This convergence can lead to a greater change in affinity than what could be expected from the normal distribution used in the shape space. This is because the affinity of a combination of CDRs is based on the affinity of the first mother cell whose descendant mutates into that combination, which can be very different from the affinity of another mother cell whose daughter B cell mutates into the same combination. This is something that may happen in a repertoire; e.g. Mathew and colleagues[1] reported an extreme case where a difference in two amino acids led to a nearly million-fold difference in the dissociation constant.

Software

Supplementary Text 2. The ABM is written in C++ [2] version 17.

The analysis of the ABM output was done on R version 4.0.3 [3] using the packages Biostrings v.2.56 [4], dplyr v.1.0.0 [5], ggplot2 v.3.3.2 [6], ggbeeswarm_ v.0.6.0 [7], igraph v.1.2.5 [8], viridis v.0.6.1 [9], readr v.1.3.1 [10], seqinr v.3.6.1 [11] and stringdist v.0.9.5.5 [12]. The analysis of the dataset from Attaf et al [13] was done on R version 4.0.3 using the packages biomaRt v. 2.44.4 [14], dplyr v.1.0.0, EDASeq v. 2.28.0 [15], GenomicRanges v.1.40.0 [16], GEOquery v. 2.56.0 [17], ggplot2 v.3.3.2, gprofiler2 v.0.2.1 [18], org.Hs.eg.db v.3.11.4 [19], plyranges v. 1.8.0 [20], Rsamtools v.2.4.0 [21], rtracklayer v.1.48.0 [22], Seurat v.4.1.0 [23] and plyr v. 1.8.6 [24].

Influx of founder cells

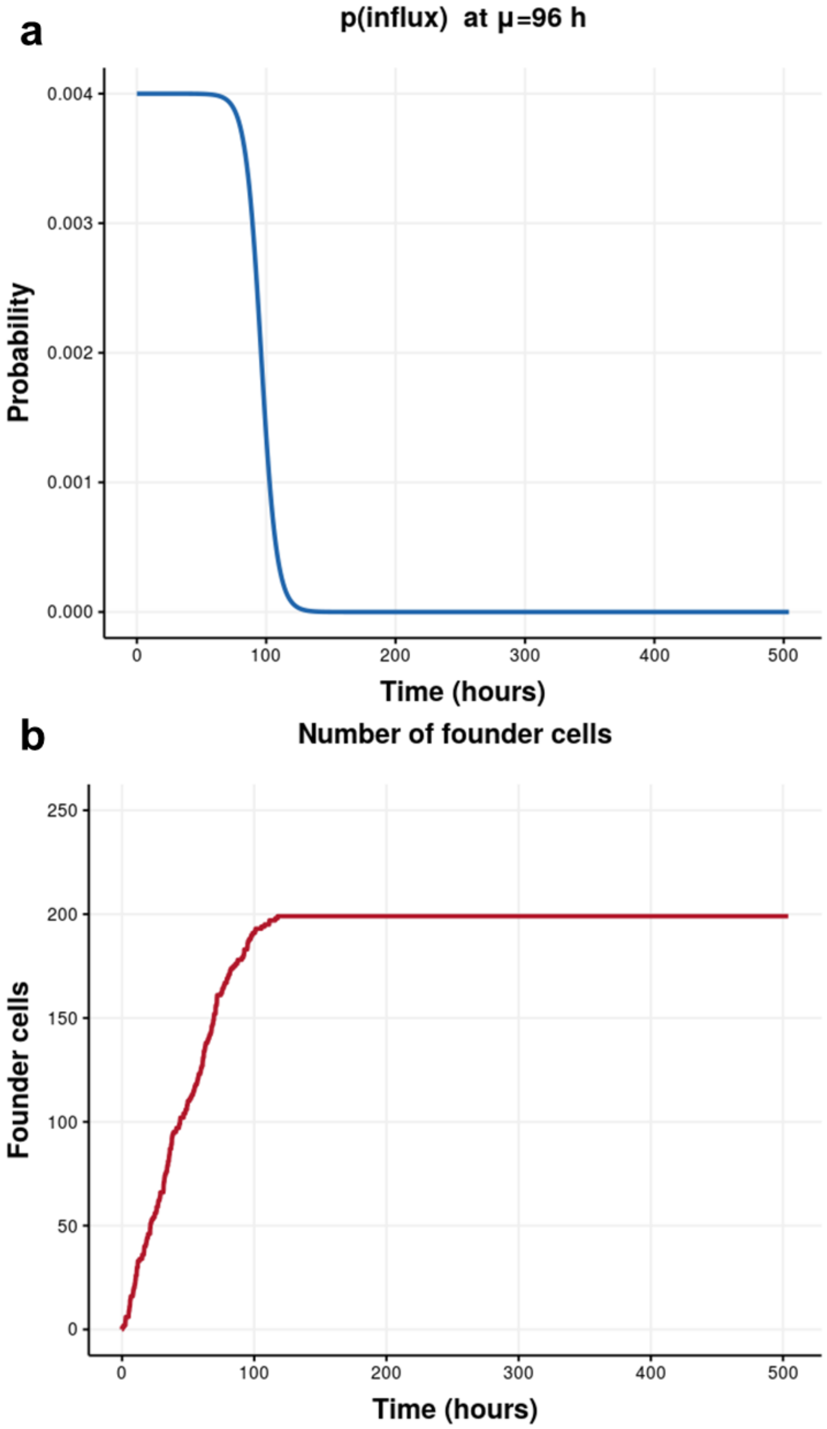

Supplementary Figure 1. (**a**) probability for a founder B cell to enter the GC reaction. (**b**) cumulative number of founder B cells that enter the GC in a single simulation, according to this probability. After approximately 96 hours the probability of influx quickly decreases until no new founder B cells enter the GC reaction.

Number of somatic hypermutations during each B cell division

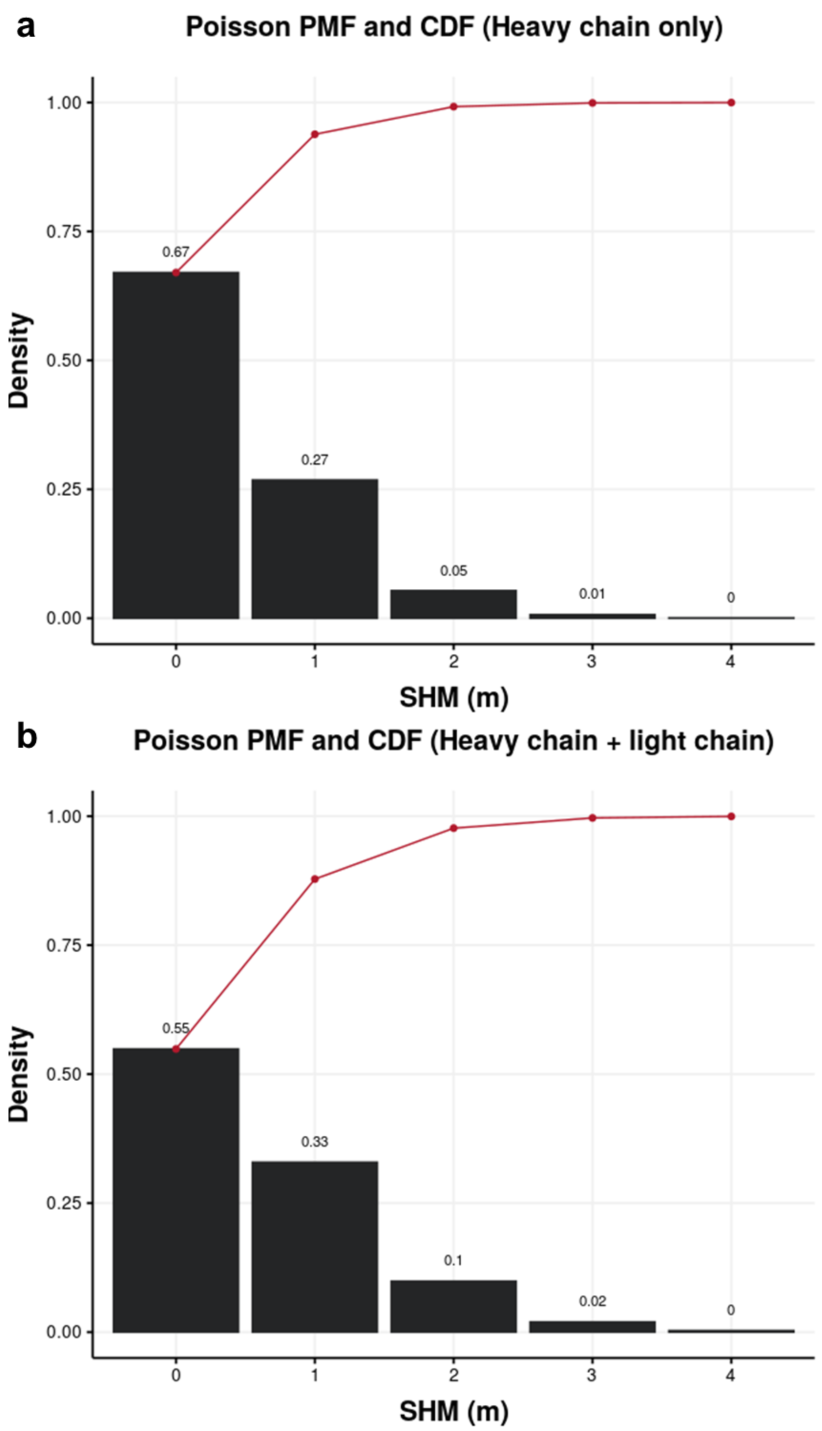

Supplementary Figure 2. Probability mass function (PMF, black bars) and cumulative distribution function (CDF, red line). (**a**) Number of BcR heavy chain somatic hypermutations assuming a heavy chain length of 400 nucleotides and a mutation rate of 1 mutation per 1000bp per B cell division[25]. Number of SHM in heavy chain is m ~ Poisson (λ=0.4) mutations per B cell division. In 33% of the B cell divisions, the BcR is mutated. (**b**) assuming a heavy chain and light chain with an approximate total length of 600 nucleotides, the BcR is mutated is 45% of the B cell divisions.

Overall GC dynamics

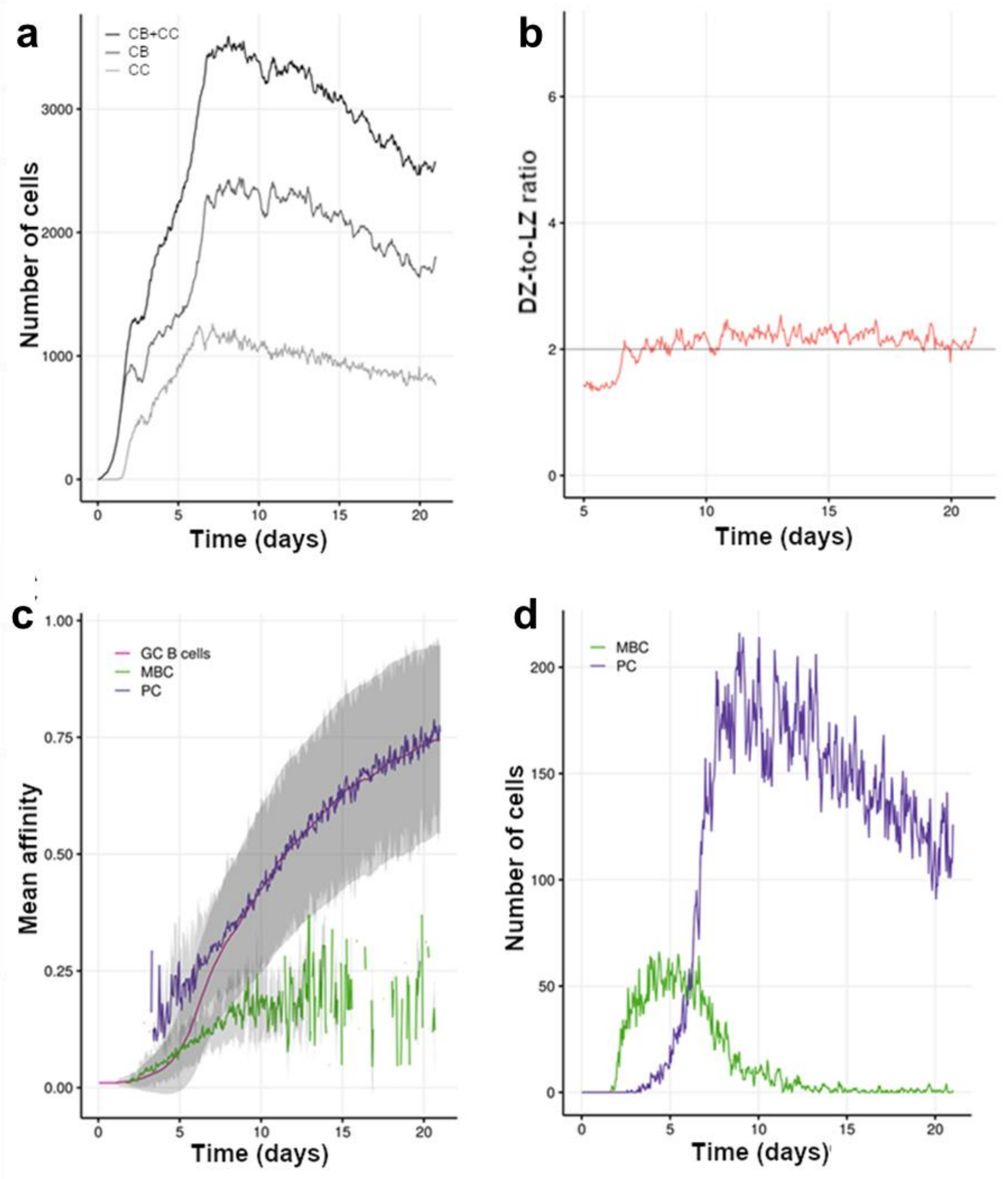

Supplementary Figure 3. Dynamics of the GC reaction observed from a single representative simulation of the eMS model of our previous computational model [26]. (**a**) Number of centrocytes and centroblasts with a peak response after about 7.5 days [27-29]. (**b**) DZ-to-LZ ratio reflecting the transzone migration rates. This ratio has shown to be approximately 2 [30]. (**c**) Affinity maturation of the GC B cells, MBCs and PCs. Lines represent the average values for that cell type. Shadowed area represents the average value plus/minus its standard deviation. The interrupted lines show the time points at which no cells of that type are present in the GC. (**d**) Number of MBCs and PCs in the GC. Most MBCs are of low affinity and produced at an early stage during the GC reaction, in contrast to PCs that are mostly of higher affinities and produced mostly after the GC peak response in agreement with experimental data from Weisel and colleagues[31].

Comparison with blood, tissue and single GC Rep-Seq data

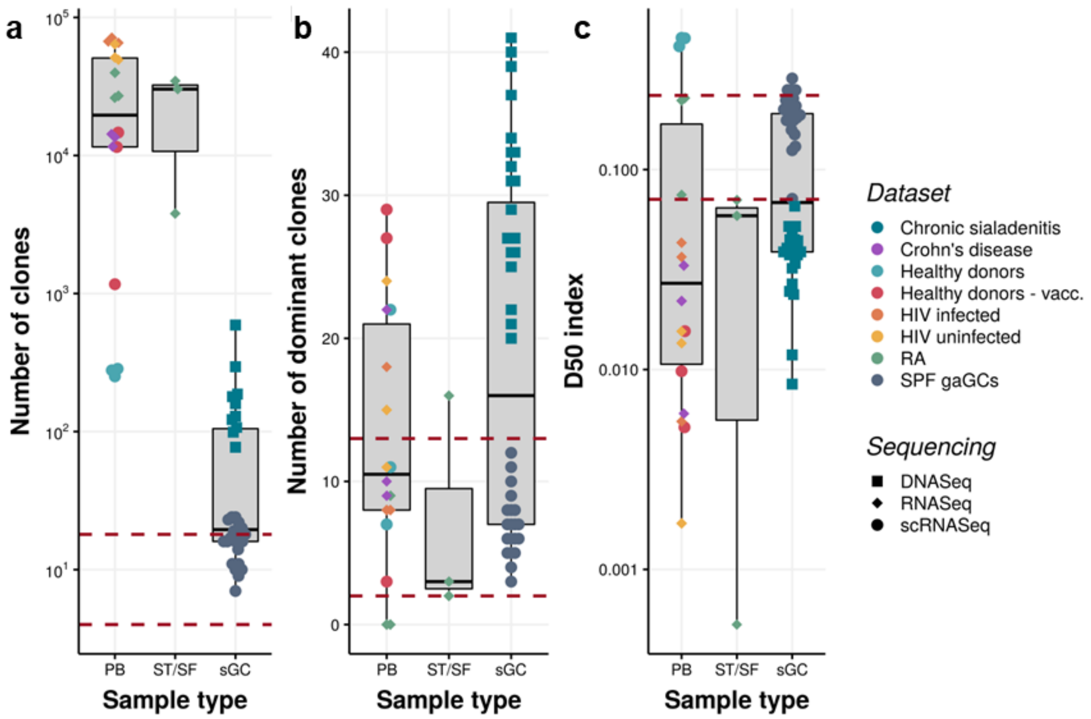

Supplementary Figure 4. Number of clones (**a**), number of dominant clones (**b**), and D50 values (**c**) determined from nine repertoire sequencing datasets. Dominant clones were defined the clones accounting for at least as 0.5% of the repertoire. The dashed red lines show the minimum and maximum values from nine simulations at day 21 of the simulation. SF: synovial fluid. ST: synovial tissue. sGC: single GC. Horizontal line: median. Box: 25th and 75th percentiles. Whiskers: 1.5 time the interquartile range.

Average size of (non)dominant clones during the GC reaction

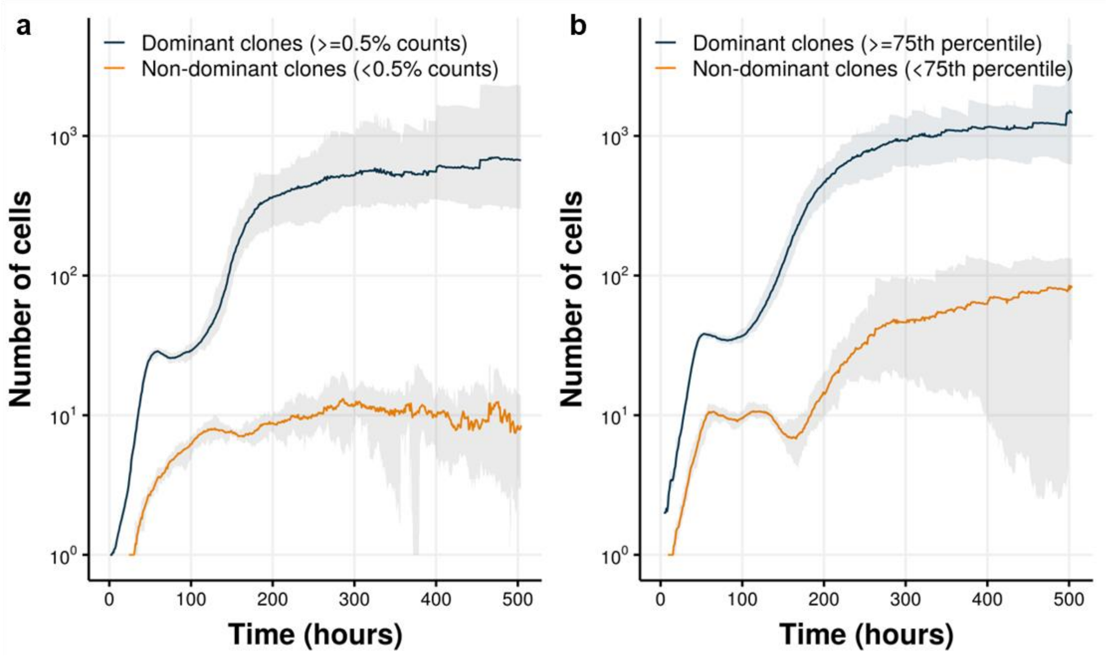

Supplementary Figure 5. Results from nine simulations showing the evolution of the mean clonal sizes of dominant clones (blue) and non-dominant clones (orange) during a 21-day GC reaction. Dominant clones were defined as those with abundances ≥0.5% of the total counts (**a**) or higher or equal than the 75th percentile of clonal abundances (**b**). The shaded area represents the minimum and maximum of the mean values obtained from the simulations. When the number of clones within a group is low, changes on its clonal composition by group switching or by elimination lead to leaps in the trends. Our simulation suggests that the definition for dominant clones should be well-considered as it can have a relevant effect on their selection. The criteria in (**a**) depends on the total number of counts while the criteria in (**b**) instead depends on the distribution of the clonal abundances. In this case (**b**) is more restrictive than (**a**).

Number of (sub)clones during GC reaction

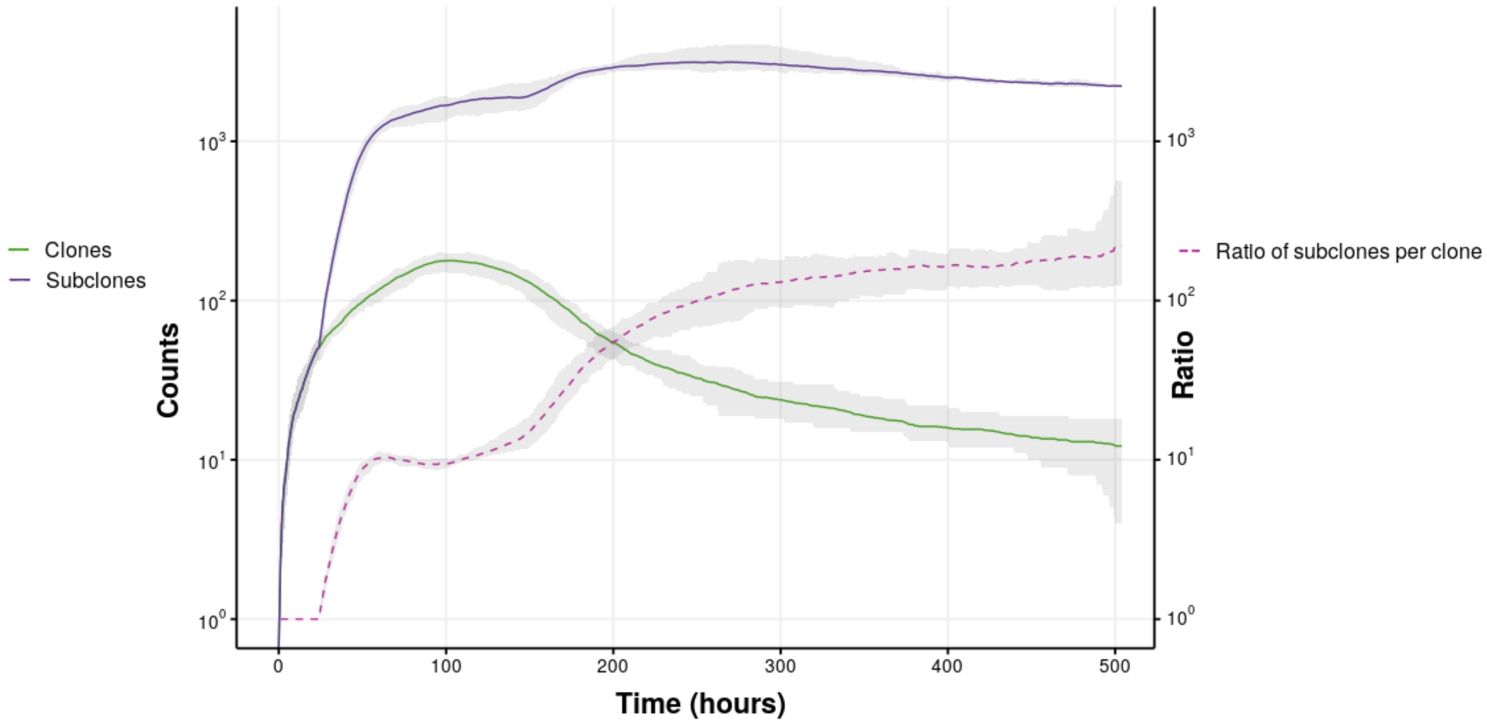

Supplementary Figure 6. Progression of the number of (sub)clones and the average ratio of subclones per clone in log10 scale during the GC reaction for nine repeated simulations. The number of clones initially increase as a result of the entry of founder B cells. Subsequently, the number of clones decrease due to clonal competition. Lines represent the average value while the shadowed area represents the interval of minimum and maximum values of the nine simulations at each time point.

Subclone dynamics

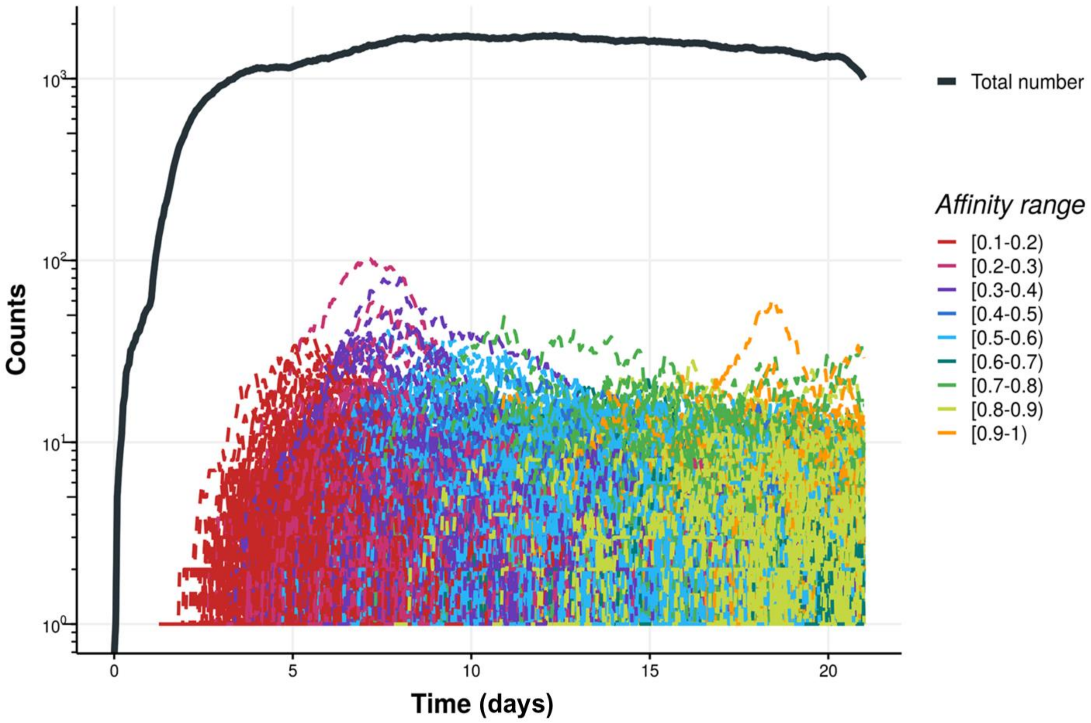

Supplementary Figure 7. Abundance and affinity of subclones, defined as B cells that share the same Fab NT sequence, during a 21-day GC reaction (representative simulation). Only subclones with a frequency larger than 3 counts at any timepoint are included in this plot. Each colored line represents a subclone with a unique BcR. When a subclone is created by SHM it will start as a single B cell that will, subsequently, proliferate. The abundance of each subclone at any timepoint does not exceed 100 copies. This is due to the fact that a mutation of one subclone B cell will, by definition, create a new subclone. Consequently, a SHM reduces the subclone count with one. This balance between proliferation and SHM prevents large subclonal frequencies. It also accounts for the relatively constant of about 2000 subclones with a frequency larger than 3 counts at any timepoint during the GC reaction (black line).

Single B cell lineage tree

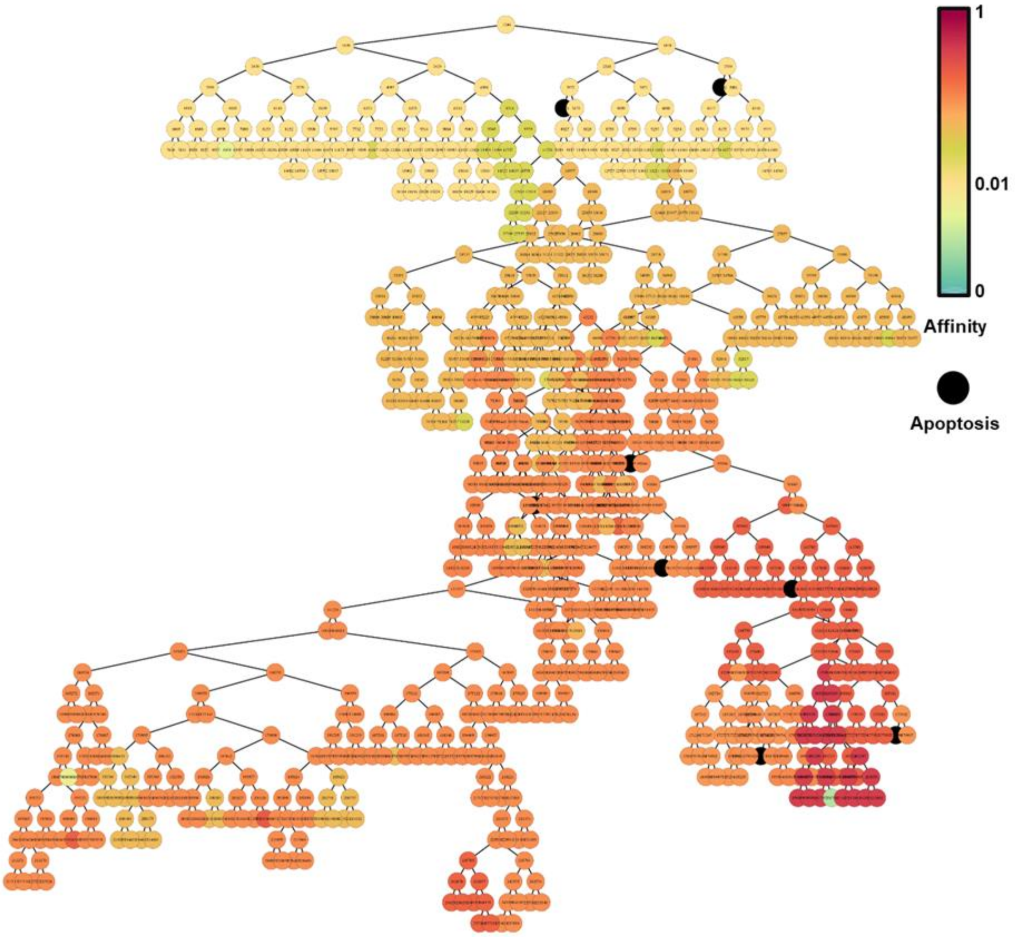

Supplementary Figure 8. Example of the division of a single unmutated founder B cell during a 21-day GC reaction. During each division two daughter B cells are generated. SHM may change the affinity of each B cell. The color of a cell denotes its affinity value; from 0 (blue: very low affinity), to 0.01 (yellow: low affinity, initial affinity of the founder cell) to 1(red: high affinity) or its functionality (black: lethal mutation, non-functional BcR). The division trees for all founder B cells are shown in the main text (Figure 5). Every cell is labeled according to their ID number.

.**Dominant clones in nine repeated simulations**

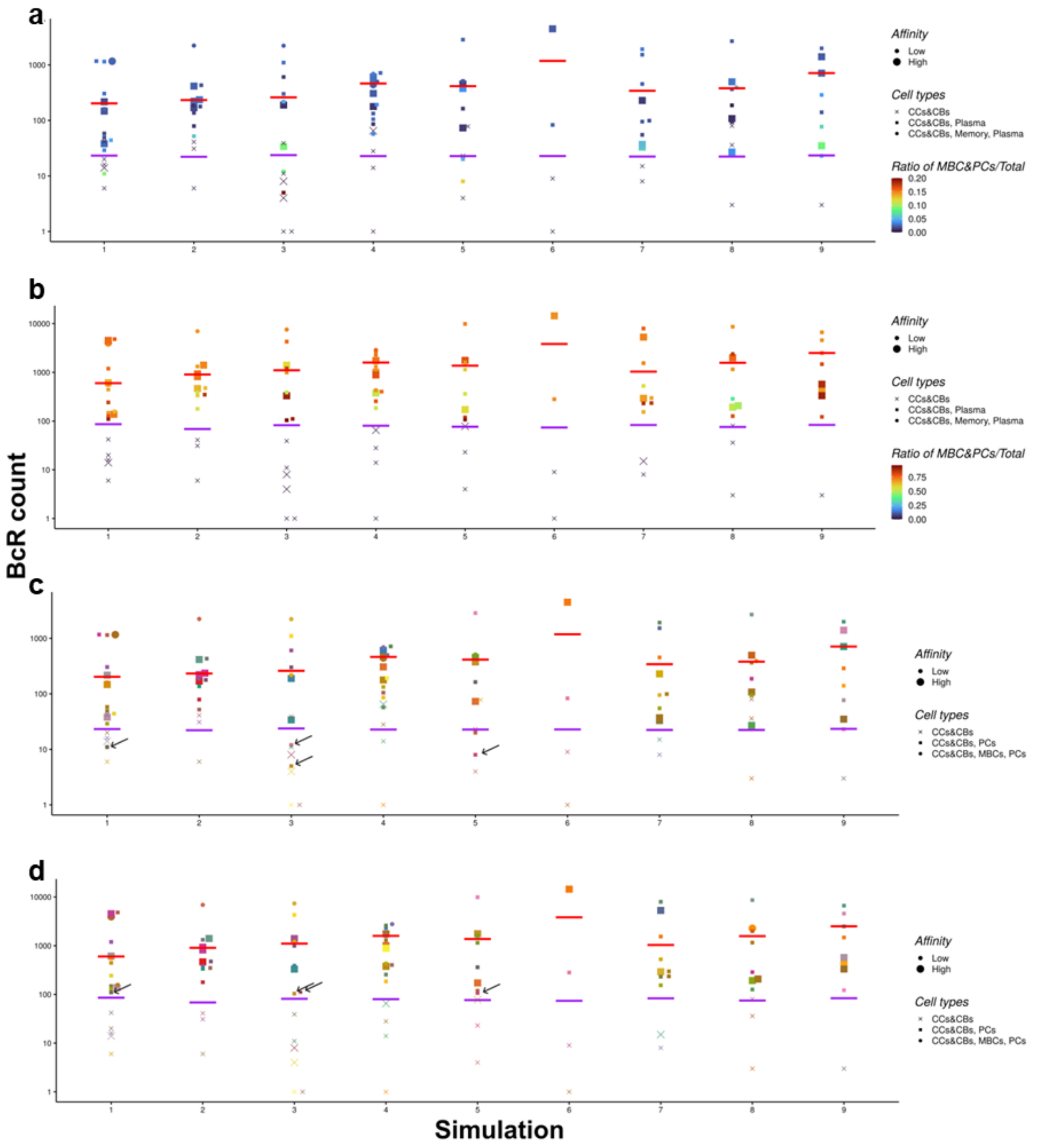

Supplementary Figure 9. (**a**, **c**) DNA-seq and (**b**, **d**) RNA-Seq repertoires at day 21 of the GC reaction generated by nine repeated simulations. Each dot represents a clone, some of which are a mixture of B cells, MBCs and/or PCs. Colors in (**a**) and (**b**) indicate the percentage of PCs within each clone, while colors in (**c**) and (**d**) represent each clone. The size of the symbol represents the median affinity of that clone (small: affinity < 75th percentile). The horizontal lines denote the 75th percentile (red) and 0.5% (purple) thresholds that define dominant clones. In (**c**) and (**d**) the arrows point to some of the clones that can be selected as dominant or not depending on the sequencing method.

Determination of BcR RNA levels

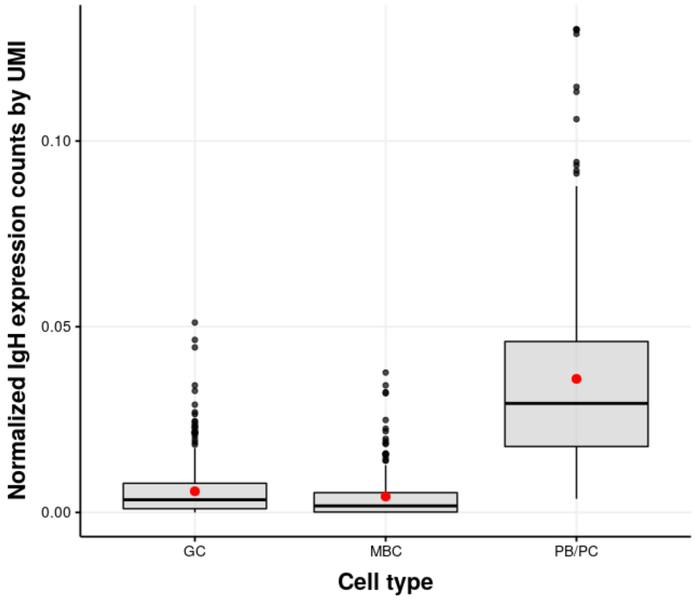

Supplementary Figure 10. Normalized IgH expression counts per cell type (GC, MBC, PB/PC). The red spots represent the mean value for each cell type. To determinate the relation between BcR levels and cell type, we processed the human tonsils datasets from Attaf and coworkers[13], following their methods, using the default Seurat workflow[23] to determinate the cell type based on expression markers. For every cell, we calculated the sum of all the IgH genes UMI counts normalized by the total cell UMI count. Then we compared the IgH expression between the different groups.

BcR counts in DNA-based vs RNA-based scenarios

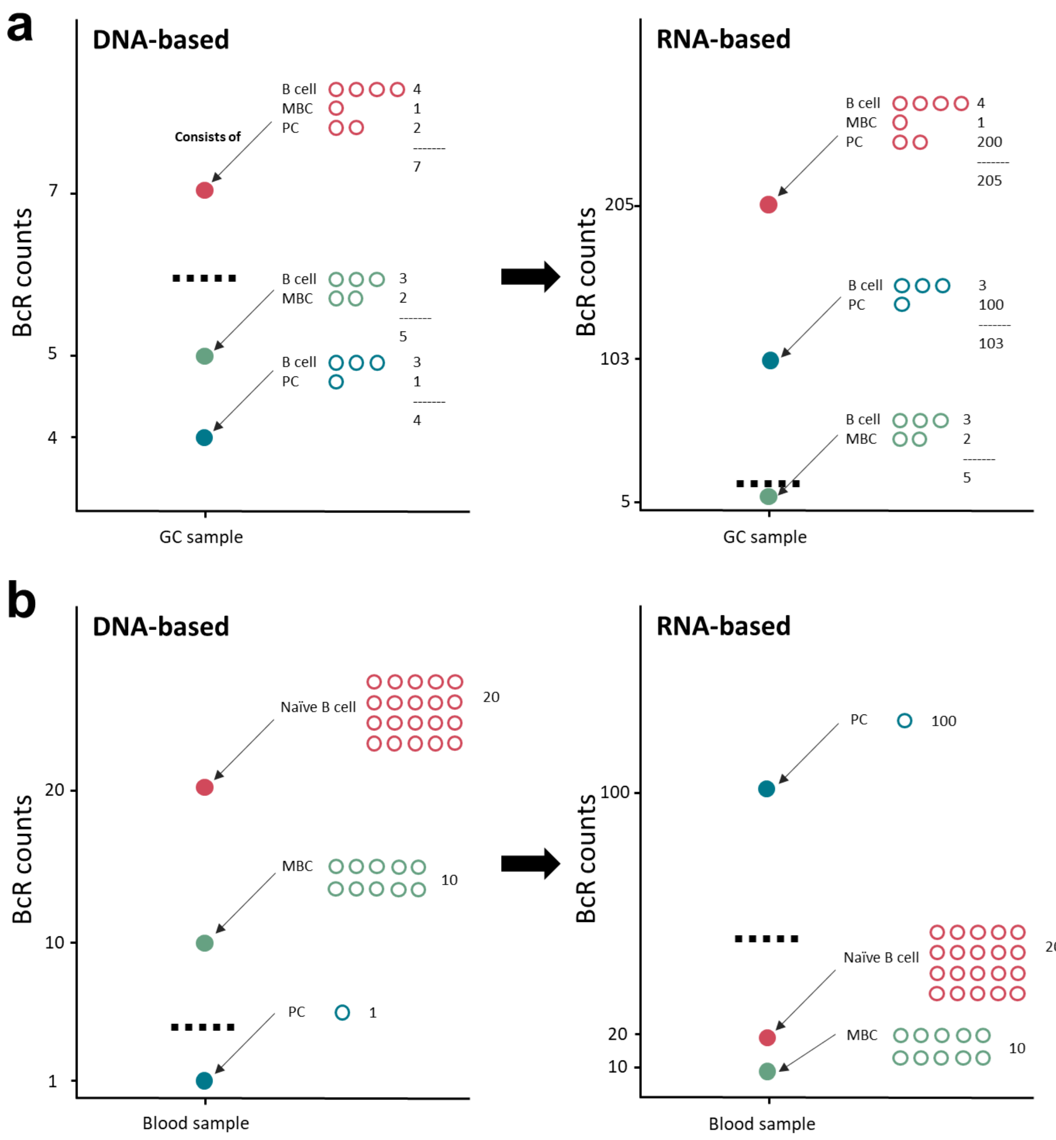

Supplementary Figure 11. Conceptual illustration of DNA-based vs RNA-based clonal size inference for three clones and a threshold for selecting the dominant clones in (**a**) a single GC sample and (**b**) peripheral blood. In the DNA-based scenario, every cell type contributes equally to the calculation of the clonal size. In the RNA-based scenario, PCs contribute up to 100 times greater than the other cell types, changing the inferred clonal sizes, distorting the results and the selection of dominant clones over the threshold. The correlation between the clonal size and its PC composition in GCs helps to reduce this change. However, in peripheral blood this correlation does not need to happen and therefore dominant clones can be wrongly selected in the RNA-based scenario.

GC ABM and gene regulatory network

Parameters for the model

Supplementary Table 1. Main parameters of the GC model. Source and additional information for these parameters related to the GC ABM can be found in previous literature[32-36]. Parameters of the ODE model of the GRN [37] are normalized by a unit of time (t_0) and concentration (C_0). p=BLIMP1, b=BCL6, r=IRF4.

Category	Parameter name	lue	Unit	Concept
Space discretization				
	**dimension**	3	dimensions	Lattice Dimensions
	**dt**	0.002	hour	Time resolution
	**tmin**	0	hour	Beginning of the simulation
	**tmax**	504	hour	Maximum duration of the simulation
	**dx**	5	µm	Lattice Constant of space grid
	**radius**	160	µm	Radius of GC
	**zoneRatioGC**	0.5		Ratio that determines the position of DZ in Germinal Center
Initialization				
	**InitialNumberSC**	300	cell	Initial Number Stromal cells
	**InitialNumberTC**	250	cell	Initial Number T-cells
	**InitialNumberCB**	0	cell	Initial Number CBs (founder cells)
	**InitialNumberFDC**	200	cell	Initial Number FDCs
	**macrophage**	6	cells/hour	Rate of macrophage transport of dead cells
	**DendriteLength**	40		Length FDC dendrites / dx (number of positions)
	**AgAmountperFDC**	3000	Antigens	Presented Antigen per FDC
	**Avogadro_constant**	6.02E+23	mol^-1	Number of Avogadro
Affinity				
	**Gamma**	2.8		Width of gaussian affinity weight function
	**eta**	2		Exponent of the hamming distance
	**expMin**	5.5	1/mol	Conversion of shape space affinity to (1/mol)
	**expMax**	9.5	1/mol	Conversion of shape space affinity to (1/mol)
	**Nmax**	9	residue	Maximum number of residues in one dimension
namic update of chemokine receptors				
	**CXCL13crit**	8.00E-11	mol	Critical CXCL13 concentration for desensitization
	**CXCL13recrit**	6.00E-11	mol	Critical CXCL13 concentration for resensitization
	**CXCL12crit**	6E-09	mol	Critical CXCL12 concentration for desensitization
	**CXCL12recrit**	4E-09	mol	Critical CXCL12 concentration for resensitization
Chemotaxis				
	**chemmax**	10		Maximum weight of chemotaxis to random polarity
	**chemosteep**	1.00E+10	mol/L	Steepness of weight reduction with chemokine gradient
	**chemohalf**	2.00E-11	L/mol	Chemokine gradient of half weight
	**chemo_dx**	5	µm	Lattice Chemokine Constant
sistentLengthTime(Cell Type)				
	**Bcell_tp**	1.5	hour	B-Cell Persistent Time average

**Bcell_tp_stddev**	0	hour	B-Cell Persistent Time stddev
**Tcell_tp**	1.7	hour	T Cell persistence time
**Tcell_tp_stddev**	0	hour	T Cell persistence time Standard deviation
**Plasmacell_tp**	0.75	hour	Plasma and Memory B Cell persistence time
**Plasmacell_tp_stddev**	-1	hour	Plasma and Memory B Cell persistence time Standard deviation
Speed(Cell Type)			
**Bcell_speed**	7.5	µM / min	B-Cell Speed
**Tcell_speed**	10	µM / min	T-Cell Speed
**Plasmacell_speed**	3	µM / min	Plasma and Memory B cell speed
ration of cell cycle phases (according to Gaussian distribution with parameters)			
**c_G1**	2.5	hour	Phase G1 of cell cycle
**c_G1_stddev**	1	hour	Stddev of phase G1 of cell cycle
**c_S**	1.5	hour	Phase S of cell cycle
**c_S_stddev**	1	hour	Stddev of phase S of cell cycle
**c_G2**	2.5	hour	Phase G2 of cell cycle
**c_G2_stddev**	1	hour	Stddev of phase G2 of cell cycle
**c_M**	0.5	hour	Phase M of cell cycle
**c_M_stddev**	1	hour	Stddev of phase M of cell cycle
Differentiation rates			
**StartDifferentiation**	72	hour	Time when differentiation can start
**difDelay**	6	hour	Delay cell differentiation after TC selection
**DeleteAgInFreshCC**	TRUE	Boolean	Retained Ag is deleted in fresh CC
**Ccdif_delay_stddev**	0	hour	Standard deviation for delay to differentiation.
**pApoCC**	0		% Casp3+ LZ cells per hr. used as (apoptosis rate)
**pApoCB**	0		% Casp3+ DZ cells per hr. used as (apoptosis rate)
**tolight**	0.1		Rate for differentiation of centroblasts to centrocytes
Dynamic number of divisions			
**pMHCdepHill**	2		p-MHC dependent division number Hill (Hill coef. n_P)
**pMHCdepMin**	1		p-MHC dependent division number Hill (Hill coef. P_Min)
**pMHCdepMax**	6		p-MHC dependent division number Hill (Hill coef. P_Max)
**pMHCdepK**	9		p-MHC dependent division number Hill (Hill coef. K_P)
**NoMutFounderCells**	FALSE	Boolean	Founder cells do not mutate
**nDiv**	12	divisions	Number of divisions of founder cells present at time 0
**nDiv_stddev**	0	divisions	Stddev of Number of divisions of founder cells present at time 0
**nDivinflow**	6	divisions	Number of divisions of founder cells that enter during the process
**rateCBinflow**	2	cells/hour	Rate of inflow of CBs (founder cells)
**smoothnessStopCBinflow**	6		Smoothness of the stop of inflow CB (-1 = no)
**timeStopCBinflow**	96	hour	Time when CB inflow stops (effectve time depends on the smoothness)
Mutation and division			
**pmutB4StartMut**	0		Probability of mutation before the start of the mutation period
**StartMutation**	24	hour	Start of the mutation period
**lambda**	0.4		Lambda value of the Poisson formula for the probability of mutations after 24 hours
**alpha_FWR1**	0.17		Probability of a mutation happening in FWR1

**alpha_FWR2**	0.17		Probability of a mutation happening in FWR2
**alpha_FWR3**	0.29		Probability of a mutation happening in FWR3
**alpha_FWR4**	0.05		Probability of a mutation happening in FWR4
**alpha_CDR1**	0.1		Probability of a mutation happening in CDR1
**alpha_CDR2**	0.03		Probability of a mutation happening in CDR2
**alpha_CDR3**	0.19		Probability of a mutation happening in CDR3
**beta_FWR1**	0.73		Probability of a R mutation in FWR1
**beta_FWR2**	0.67		Probability of a R mutation in FWR2
**beta_FWR3**	0.73		Probability of a R mutation in FWR3
**beta_FWR4**	0.6		Probability of a R mutation in FWR4
**gamma_CDR1**	0.79		Probability of a R mutation in CDR1
**gamma_CDR2**	0.76		Probability of a R mutation in CDR2
**gamma_CDR3**	0.75		Probability of a R mutation in CDR3
**delta**	0.5		Probability of a lethal R mutation in the FWRs, always leading to apoptosis
**polarityIndex**	1		Assymetric Distribution of Ag
**widthPI**	0.04		Coefficient of variation arround Polarity Index
**pDivideAgAssymetric**	0.72		Probability to divide Ag assymetrically to daughter B-cell
Selection steps			
**testDelay**	0.02	hour	Time gap between TFHC-CC binding tests
**collectionFDCperiod**	0.7	hour	Duration of CC collection of Antigen by serial encounters with FDC
**agSaturation**	20	threshold	Ag saturation per FDC fragment in units of threshold. 1:constant
**pSel**	0.04		Probability to be selected by FDC
**tcTime**	0.6	hour	Duration of CC-Tc contact
**tcRescueTime**	0.5	hour	Minimum duration of TC-CC-polarization for CC-rescue
**Ag_threshold**	1.00E-08	mol	Threshold Ag-concentration for binding CC
Antibody/BcR			
**kon**	1.00E+06	1/(mol*h)	k_on for building immune complex
**koff**	0.001	1/s	k_off for dissociation of immune complex (in /s):
**BCR_length**	4	dimension	Dimensions in the spape space
ODE system			
μ_p	1.00E-6	C_0/t_0	Basal transcription rate
μ_b	2	C_0/t_0	Basal transcription rate
μ_r	0.1	C_0/t_0	Basal transcription rate
σ_p	9	C_0/t_0	Maximum induced transcription rate
σ_b	100	C_0/t_0	Maximum induced transcription rate
σ_r	2.6	C_0/t_0	Maximum induced transcription rate
κ_p	1	C_0	Dissociation constant
κ_b	1	C_0	Dissociation constant
κ_r	1	C_0	Dissociation constant
λ_p	1	$1/t_0$	Degradation rate
λ_b	1	$1/t_0$	Degradation rate
λ_r	1	$1/t_0$	Degradation rate
**bcr0**	1	$1/t_0$	Maximum BCR signal

**cd0**	50	C_0/t_0	Maximum CD40 signal
$\mathbf{C_0}$	1.00E-8	M	Concentration unit
$\mathbf{t_0}$	4	h	Time unit

Supplementary Table 2. Results at day 21 from nine repeated simulations.

Simulation	Clones	Dominant clones				D50	
		DNA-based		RNA-based			
		75th percentile	0.5%	75th percentile	0.5%	DNA-based	RNA-based
**1**	18	5	13	5	13	0.11	0.11
**2**	14	4	13	4	11	0.07	0.07
**3**	15	4	8	4	9	0.13	0.13
**4**	17	4	15	4	13	0.24	0.24
**5**	11	3	8	3	9	0.09	0.09
**6**	4	1	2	1	2	0.25	0.25
**7**	11	3	9	3	9	0.18	0.18
**8**	11	3	10	3	9	0.09	0.09
**9**	9	2	7	2	8	0.22	0.22
**Average**	**12**	**3**	**9**	**3**	**9**	**0.15**	**0.15**

References

1 Mathew, N. R. *et al.* Single-cell BCR and transcriptome analysis after influenza infection reveals spatiotemporal dynamics of antigen-specific B cells. *Cell Rep* **35**, 109286, doi:10.1016/j.celrep.2021.109286 (2021).

2 The C++ programming language (Addison-Wesley, 1985).

3 R: A language and environment for statistical computing. R Foundation for Statistical Computing (2021).

4 Biostrings: Efficient manipulation of biological strings (2021).

5 dplyr: A Grammar of Data Manipulation (2021).

6 Wickham, H. *ggplot2: Elegant Graphics for Data Analysis*. (Springer-Verlag New York, 2016).

7 ggbeeswarm: Categorical Scatter (Violin Point) Plots (2017).

8 Csardi, G. & Nepusz, T. The igraph software package for complex network research. *InterJournal* **Complex Systems**, 1695 (2006).

9 Garnier *et al.* viridis - Colorblind-Friendly Color Maps for R. doi:https://doi.org/10.5281/zenodo.4679424 (2021).

10 readr: Read Rectangular Text Data (2022).

11 Charif, D. & Lobry, J. R. in *Structural approaches to sequence evolution: Molecules, networks, populations Biological and Medical Physics, Biomedical Engineering* (eds U. Bastolla, M. Porto, H.E. Roman, & M. Vendruscolo) 207-232 (Sprinter Verlag, 2007).

12 van der Loo, M. P. J. The stringdist package for approximate string matching. *The R Journal* **6**, 111-122 (2014).

13 Attaf, N. *et al.* FB5P-seq: FACS-Based 5-Prime End Single-Cell RNA-seq for Integrative Analysis of Transcriptome and Antigen Receptor Repertoire in B and T Cells. *Front Immunol* **11**, 216, doi:10.3389/fimmu.2020.00216 (2020).

14 Durinck, S. *et al.* BioMart and Bioconductor: a powerful link between biological databases and microarray data analysis. *Bioinformatics* **21**, 3439–3440 (2005).

15 Risso, D., Schwartz, K., Sherlock, G. & Dudoit, S. GC-content normalization for RNA-Seq data. *BMC bioinformatics* **12**, 480, doi:10.1186/1471-2105-12-480 (2011).

16 Lawrence, M. *et al.* Software for computing and annotating genomic ranges. *PLoS Comput Biol* **9**, e1003118, doi:10.1371/journal.pcbi.1003118 (2013).

17 Davis, S. & Meltzer, P. GEOquery: a bridge between the Gene Expression Omnibus (GEO) and BioConductor. *Bioinformatics* **14**, 1846–1847 (2007).

18 Kolberg, L., Raudvere, U., Kuzmin, I., Vilo, J. & Peterson, H. gprofiler2 -- an R package for gene list functional enrichment analysis and namespace conversion toolset g:Profiler. *F1000Res* **9**, doi:10.12688/f1000research.24956.2 (2020).

19 org.Hs.eg.db: Genome wide annotation for Human.

20 Lee, S., Cook, D. & Lawrence, M. plyranges: a grammar of genomic data transformation. *Genome Biol* **20**, 4, doi:10.1186/s13059-018-1597-8 (2019).

21 Rsamtools: Binary alignment (BAM), FASTA, variant call (BCF), and tabix file import (2021).

22 Lawrence, M., Gentleman, R. & Carey, V. rtracklayer: an R package for interfacing with genome browsers. *Bioinformatics* **25**, 1841-1842, doi:10.1093/bioinformatics/btp328 (2009).
23 Hao, Y. *et al.* Integrated analysis of multimodal single-cell data. *Cell* **184**, 3573-3587 e3529, doi:10.1016/j.cell.2021.04.048 (2021).
24 Wickham, H. The Split-Apply-Combine Strategy for Data Analysis. *Journal of Statistical Software* **40**, 1-29 (2011).
25 Reshetova, P. *et al.* Computational Model Reveals Limited Correlation between Germinal Center B-Cell Subclone Abundancy and Affinity: Implications for Repertoire Sequencing. *Front Immunol* **8**, 221, doi:10.3389/fimmu.2017.00221 (2017).
26 Merino Tejero, E. *et al.* Multiscale Modeling of Germinal Center Recapitulates the Temporal Transition From Memory B Cells to Plasma Cells Differentiation as Regulated by Antigen Affinity-Based Tfh Cell Help. *Front Immunol* **11**, 620716, doi:10.3389/fimmu.2020.620716 (2020).
27 Liu, Y. J., Zhang, J., Lane, P. J., Chan, E. Y. & MacLennan, I. C. Sites of specific B cell activation in primary and secondary responses to T cell-dependent and T cell-independent antigens. *Eur J Immunol* **21**, 2951-2962, doi:10.1002/eji.1830211209 (1991).
28 Hollowood, K. & Macartney, J. Cell kinetics of the germinal center reaction--a stathmokinetic study. *Eur J Immunol* **22**, 261-266, doi:10.1002/eji.1830220138 (1992).
29 Wittenbrink, N., Klein, A., Weiser, A. A., Schuchhardt, J. & Or-Guil, M. Is there a typical germinal center? A large-scale immunohistological study on the cellular composition of germinal centers during the hapten-carrier-driven primary immune response in mice. *J Immunol* **187**, 6185-6196, doi:10.4049/jimmunol.1101440 (2011).
30 Victora, G. D. *et al.* Germinal center dynamics revealed by multiphoton microscopy with a photoactivatable fluorescent reporter. *Cell* **143**, 592-605, doi:10.1016/j.cell.2010.10.032 (2010).
31 Weisel, F. J., Zuccarino-Catania, G. V., Chikina, M. & Shlomchik, M. J. A Temporal Switch in the Germinal Center Determines Differential Output of Memory B and Plasma Cells. *Immunity* **44**, 116-130, doi:10.1016/j.immuni.2015.12.004 (2016).
32 Meyer-Hermann, M. & Beyer, T. The type of seeder cells determines the efficiency of germinal center reactions. *Bull Math Biol* **66**, 125-141, doi:10.1016/j.bulm.2003.08.003 (2004).
33 Meyer-Hermann, M. *et al.* A theory of germinal center B cell selection, division, and exit. *Cell Rep* **2**, 162-174, doi:10.1016/j.celrep.2012.05.010 (2012).
34 Meyer-Hermann, M. A mathematical model for the germinal center morphology and affinity maturation. *J Theor Biol* **216**, 273-300, doi:10.1006/jtbi.2002.2550 (2002).
35 Meyer-Hermann, M. E. & Maini, P. K. Interpreting two-photon imaging data of lymphocyte motility. *Phys Rev E Stat Nonlin Soft Matter Phys* **71**, 061912, doi:10.1103/PhysRevE.71.061912 (2005).
36 Robert, P. A., Rastogi, A., Binder, S. C. & Meyer-Hermann, M. in *Germinal Centers: Methods and Protocols* Vol. 1623 303-334 (2017).

37 Martinez, M. R. *et al.* Quantitative modeling of the terminal differentiation of B cells and mechanisms of lymphomagenesis. *Proc Natl Acad Sci U S A* **109**, 2672-2677, doi:10.1073/pnas.1113019109 (2012).
