# Supplementary material for: Understanding repertoire sequencing data through a multiscale computational model of the germinal center
Source: NPJ Syst Biol Appl. 2023 Mar 16;9:8. doi: 10.1038/s41540-023-00271-y (PMC10019394; doi:10.1038/s41540-023-00271-y)
Supplement: Supplementary file 2 — Reporting Summary [file 41540_2023_271_MOESM2_ESM.pdf]

nature portfolio

Corresponding author(s): Antoine van Kampen

Last updated by author(s): Feb 15, 2023

Reporting Summary

Nature Portfolio wishes to improve the reproducibility of the work that we publish. This form provides structure for consistency and transparency in reporting. For further information on Nature Portfolio policies, see our Editorial Policies and the Editorial Policy Checklist.

Statistics

For all statistical analyses, confirm that the following items are present in the figure legend, table legend, main text, or Methods section.

n/a	Confirmed	
☐	☒	The exact sample size (*n*) for each experimental group/condition, given as a discrete number and unit of measurement
☐	☒	A statement on whether measurements were taken from distinct samples or whether the same sample was measured repeatedly
☒	☐	The statistical test(s) used AND whether they are one- or two-sided<br>*Only common tests should be described solely by name; describe more complex techniques in the Methods section.*
☒	☐	A description of all covariates tested
☒	☐	A description of any assumptions or corrections, such as tests of normality and adjustment for multiple comparisons
☐	☒	A full description of the statistical parameters including central tendency (e.g. means) or other basic estimates (e.g. regression coefficient) AND variation (e.g. standard deviation) or associated estimates of uncertainty (e.g. confidence intervals)
☒	☐	For null hypothesis testing, the test statistic (e.g. *F*, *t*, *r*) with confidence intervals, effect sizes, degrees of freedom and *P* value noted<br>*Give P values as exact values whenever suitable.*
☒	☐	For Bayesian analysis, information on the choice of priors and Markov chain Monte Carlo settings
☒	☐	For hierarchical and complex designs, identification of the appropriate level for tests and full reporting of outcomes
☒	☐	Estimates of effect sizes (e.g. Cohen's *d*, Pearson's *r*), indicating how they were calculated

Our web collection on statistics for biologists contains articles on many of the points above.

Software and code

Policy information about availability of computer code

Data collection	The GC ABM model used in the manuscript is written in C++ version 17 and is published in https://github.com/EDS-Bioinformatics-Laboratory/GC_ABM_SHM_network
Data analysis	The analysis of the ABM output was done on R version 4.0.3 using the packages Biostrings v.2.56, dplyr v.1.0.0, ggplot2 v.3.3.2, ggbeeswarm_v.0.6.0, igraph v.1.2.5, viridis v.0.6.1, readr v.1.3.1, seqinr v.3.6.1 and stringdist v.0.9.5.5. The analysis of the dataset from Attaf et al was done on R version 4.0.3 using the packages biomaRt v. 2.44.4, dplyr v.1.0.0, EDASeq v. 2.28.0, GenomicRanges v.1.40.0, GEOquery v. 2.56.0, ggplot2 v.3.3.2, gprofiler2 v.0.2.1, org.Hs.eg.db v.3.11.4, plyranges v. 1.8.0, Rsamtools v.2.4.0, rtracklayer v.1.48.0, Seurat v.4.1.0 and plyr v. 1.8.6.

For manuscripts utilizing custom algorithms or software that are central to the research but not yet described in published literature, software must be made available to editors and reviewers. We strongly encourage code deposition in a community repository (e.g. GitHub). See the Nature Portfolio guidelines for submitting code & software for further information.

Data

Policy information about availability of data

All manuscripts must include a data availability statement. This statement should provide the following information, where applicable:

- Accession codes, unique identifiers, or web links for publicly available datasets
- A description of any restrictions on data availability
- For clinical datasets or third party data, please ensure that the statement adheres to our policy

Simulation parameters and setup details are available in Supplementary Table 1 and GitHub [https://github.com/EDS-Bioinformatics-Laboratory/GC_ABM_SHM_network]. Individual simulation results generated and analyzed during the current study are available at 10.5281/zenodo.7642721.
Dataset (ii), analysed during the current study is available at NCBI GEO under the accession number GSE196820. Datasets (v) and (vi), analysed during the current study, are available under the accession number PRJNA822925. Dataset (viii), analysed during the current study, is available from J.G, and its processed data is deposited on the VDJ server under UUID 8899006209436478995-242ac118-0001-012. The remaining datasets are available from their original publications.

Human research participants

Policy information about studies involving human research participants and Sex and Gender in Research.

Reporting on sex and gender	*Use the terms sex (biological attribute) and gender (shaped by social and cultural circumstances) carefully in order to avoid confusing both terms. Indicate if findings apply to only one sex or gender; describe whether sex and gender were considered in study design whether sex and/or gender was determined based on self-reporting or assigned and methods used. Provide in the source data disaggregated sex and gender data where this information has been collected, and consent has been obtained for sharing of individual-level data; provide overall numbers in this Reporting Summary. Please state if this information has not been collected. Report sex- and gender-based analyses where performed, justify reasons for lack of sex- and gender-based analysis.*
Population characteristics	*Describe the covariate-relevant population characteristics of the human research participants (e.g. age, genotypic information, past and current diagnosis and treatment categories). If you filled out the behavioural & social sciences study design questions and have nothing to add here, write "See above."*
Recruitment	*Describe how participants were recruited. Outline any potential self-selection bias or other biases that may be present and how these are likely to impact results.*
Ethics oversight	*Identify the organization(s) that approved the study protocol.*

Note that full information on the approval of the study protocol must also be provided in the manuscript.

Field-specific reporting

Please select the one below that is the best fit for your research. If you are not sure, read the appropriate sections before making your selection.

☒ Life sciences ☐ Behavioural & social sciences ☐ Ecological, evolutionary & environmental sciences

For a reference copy of the document with all sections, see nature.com/documents/nr-reporting-summary-flat.pdf

Life sciences study design

All studies must disclose on these points even when the disclosure is negative.

Sample size	We have worked with 10 repertoire datasets from public repositories, over which we have done descriptive analysis. Sample sizes for each of them were chosen according to their category. For each blood and tissue repertoire, we selected three different random samples that included each relevant subtype in order to represent the variety of the dataset. For the single GC repertoires, as they were more relevant for the study, we selected all available samples on each dataset.
Data exclusions	For the blood and tissue repertoire datasets, as we considered it was enough to describe their general behaviour, we randomly selected subsets from each to include in the analysis.
Replication	We have analyzed publicly available repertoire datasets using public packages and custom code. Every time we have reanalyzed these samples, the results have been replicated.
Randomization	Randomization is not relevant to our study. The repertoire datasets come from different studies, we are not doing statistical testing with them, and even in some cases we randomly subselect some samples from the blood and tissue samples.
Blinding	Blinding is not relevant to our study. Regarding the repertoire datasets, the data collection happened in other groups/publications, and we analyzed the data in bulk without prior objective, merely representing the results.

Reporting for specific materials, systems and methods

We require information from authors about some types of materials, experimental systems and methods used in many studies. Here, indicate whether each material, system or method listed is relevant to your study. If you are not sure if a list item applies to your research, read the appropriate section before selecting a response.

Materials & experimental systems

n/a	Involved in the study
☒	☐ Antibodies
☒	☐ Eukaryotic cell lines
☒	☐ Palaeontology and archaeology
☒	☐ Animals and other organisms
☒	☐ Clinical data
☒	☐ Dual use research of concern

Methods

n/a	Involved in the study
☒	☐ ChIP-seq
☒	☐ Flow cytometry
☒	☐ MRI-based neuroimaging
